# Hsa_circ_0021727 (circ-CD44) promotes ESCC progression by targeting miR-23b-5p to activate the TAB1/NFκB pathway

**DOI:** 10.1038/s41419-022-05541-x

**Published:** 2023-01-06

**Authors:** Fan Meng, Xiaokang Zhang, Yanting Wang, Jie Lin, Yulin Tang, Guisheng Zhang, Binqiang Qiu, Xingdu Zeng, Weiyou Liu, Xin He

**Affiliations:** 1 Digestive System Department, The First Affiliated Hospital of Gannan Medical University, Ganzhou, China; 2 Jiangxi Provincial Branch of China Clinical Medical Research Center for Geriatric Diseases, The First Affiliated Hospital of Gannan Medical University, Ganzhou, China; 3 Department of Respiratory and Critical Illness Medicine, The First Affiliated Hospital of Gannan Medical University, Ganzhou, China

**Keywords:** Cancer epigenetics, Tumour biomarkers

## Abstract

Esophageal squamous cell carcinoma (ESCC) is characterized by high morbidity and mortality. Circular RNAs (circRNAs) play an important role in tumor progression. We discovered an aberrantly expressed circRNA (hsa_circ_0021727) in patients with ESCC. However, the mechanism of action of hsa_circ_0021727 in tumors is unclear. The present study aimed to investigate the biological role of hsa_circ_0021727 and its mechanism in ESCC progression. We screened for the expression of hsa_circ_0021727 in ESCC patients. Patients with ESCC with high expression of hsa_circ_0021727 had shorter survival than those with low expression. Hsa_circ_0021727 promoted the proliferation, invasion, and migration of ESCC cells. However, miR-23b-5p inhibited this ability of hsa_circ_0021727. MiR-23b-5p acts by targeting TAK1-binding protein 1 (TAB1). Upregulation of TAB1 can activate the nuclear factor kappa B (NFκB) pathway. Hsa_circ_0021727 promoted ESCC progression by activating TAB1/NFκB pathway by sponging miR-23b-5p. In addition, in vivo experiments also confirmed that hsa_circ_0021727 could promote the proliferation, invasion, and migration of ESCC cells. In short, hsa_circ_0021727 promotes ESCC progression by targeting miR-23b-5p to activate the TAB1/NFκB pathway. These findings might provide potential targets to treat ESCC.

## Introduction

There were 604,100 new cases of esophageal cancer worldwide in 2020, accounting for 3.1% of all cancer cases and ranking eighth; the number of deaths reached 544,076, accounting for 5.5%, ranking sixth [1, 2]. Esophageal squamous cell carcinoma (ESCC) and esophageal adenocarcinoma are the two main pathological subtypes of esophageal cancer, and ESCC is predominant in China [3]. Currently, the vast majority of patients with ESCC are in the middle and late stages when they are diagnosed, losing the chance of a cure, and the 5-year survival rate is less than 20% [4, 5]. The etiology of ESCC remains unclear. Previous studies have shown that chronic esophageal injury, esophageal inflammation, and changes in gene expression caused by a high-temperature diet or alcohol consumption might be associated with ESCC [6–8]. In recent years, in-depth research on the pathogenesis of ESCC has shown that finding more reliable targets has become an extremely urgent task.

After the completion of the Human Genome Project, it was found that only 2% of the genes function to stably transcribe and translate proteins, while the vast majority are noncoding RNAs (ncRNAs) [9, 10]. Circular RNA (circRNA) is a special kind of ncRNA molecule, which is involved in the pathogenesis of various diseases. In contrast to traditional linear RNAs, circRNA molecules have a closed ring structure, which is not affected by RNA exonuclease, thus their expression is more stable and they are not easily degraded. CircRNAs play an important role in malignant tumors, cardiovascular and cerebrovascular diseases, diabetes, and other diseases [11–13]. A large number of circRNAs are widely involved in various stages of cancer occurrence and development. ESCC was also found to be highly correlated with the expression of circRNAs. For instance, nuclear genome-derived circular RNA circPUM1 is localized in mitochondria and regulates oxidative phosphorylation in ESCC [14]. CircRNA-DOPEY2 was found to enhance chemosensitivity of esophageal cancer cells by inhibiting cytoplasmic polyadenylation element binding protein 4 (CPEB4)-mediated translation of *MCL1* (encoding myeloid cell leukemia 1) [15]. Circular RNA hsa_circ_0000277 sequesters miR-4766-5p to upregulate laminin subunit alpha 1 (LAMA1) and promote esophageal carcinoma progression [16]. CircGSK3β is thought to promote the metastasis of ESCC by enhancing β-catenin signaling [17]. Our understanding of the involvement of circRNAs is still at a very superficial stage. Therefore, the molecular mechanism of circRNAs in ESCC deserves further in-depth study.

Among the reported molecular mechanisms of circRNAs, the most common one is to competitively bind to certain microRNAs (miRNAs), acting as an “sponge” for miRNAs, preventing them from interacting with their target mRNAs via the 3′ untranslated region, thereby indirectly regulating the expression of the downstream target genes of miRNAs [18, 19].

MiRNAs, a class of noncoding single-stranded RNA molecules of about 22 nucleotides in length encoded by endogenous genes, are involved in post-transcriptional gene expression regulation in animals and plants [20]. These small RNAs have a wide range of roles in the regulation of eukaryotic gene expression. Their mechanisms include cleaving mRNA molecules of their target genes and inhibiting their translation [21, 22]. MiR-23b have been identified to act both as oncogenes and as tumor suppressors [23]. MiR-23b-5p and miR-23b-3p evolved from miR-23b.They played an important role in the occurrence and development of cancer.MiR-23b-3p was found to induce proliferation and metastasis of ESCC cells by inhibiting EBF3 [24]. MiR-23b-3p also plays a key role in pancreatic cancer progression as a negative regulator of the long noncoding RNA *KTN1-AS1* [25]. MiR-23b-5p was found to be a potential target for the treatment of acute myeloid leukemia [26]. Long noncoding RNA *CCAT2* might act as a competitive endogenous RNA (ceRNA) to regulate *FOXC1* (encoding forkhead box C1) expression by competitively binding miR-23b-5p in lung adenocarcinoma [27]. However,the relationship between miR-23b-5p and ESCC remains unclear.In this paper, we explore the mechanism of miR-23b-5p in ESCC.

To explore the role of circRNAs in the occurrence and development of ESCC, we detected the expression of circRNAs in ESCC patient tissues, screened circRNAs that might be targets for the early diagnosis and treatment of ESCC, and clarified their possible molecular mechanisms.

## Materials and methods

### Clinical tissue specimens

We collected eight ESCC tissue samples. All samples were from the First Affiliated Hospital of Gannan Medical College, collected from January to December in 2019. After surgery, the tissue specimens were frozen in liquid nitrogen immediately. None of these patients had received radiation or chemotherapy. Patients were selected based on a clear pathological diagnosis of early stage (Stages IA-IIIA) ESCC. All patients’ follow-up records were collected from January 2006 to July 2015. All patients were well informed, the processes were approved by the Ethics Committee of The First Affiliated Hospital of Gannan Medical University, and written informed consent was obtained from each patient.

Two tissue microarrays were used to evaluate the expression of hsa_circ_0021727 using in situ hybridization (ISH). The tissue microarrays were purchased from Shanghai Outdo Biotech Co., Ltd. (Shanghai, China) and contained approximately 170 pairs of ESCC samples and 100 pairs of their para-carcinoma tissues.

### CircRNA microarray

To screen out circRNAs that can be used as targets, we performed circRNA microarray hybridization and data analysis on the three pairs of tissue samples collected. Total RNA was digested with RNase R (Epicentre, Madison, WI, USA) to remove linear RNAs and enrich circRNAs. Then, the enriched circRNAs were amplified and transcribed to fluorescent complimentary RNA (cRNA) utilizing a random priming method (Arraystar Super RNA Labeling Kit; Arraystar, Rockville, MD USA). The labeled cRNAs were hybridized to an Arraystar Human circRNA Array (8 × 15 K, Arraystar). After washing the slides, the arrays were scanned using an Agilent G2505C scanner (Agilent, Santa Clara, CA, USA). Agilent Feature Extraction software (version 11.0.1.1) was used to analyze the acquired array images.

### CircRNA in situ hybridization (RNA-ISH)

All experimental steps were performed according to the standard procedures of ISH [28]. The tissue microarray was deparaffinized, rehydrated through an ethanol gradient, and then treated with 20 μg/mL proteinase K (Roche Diagnostics, Indianapolis, IN, USA). Then, it was fixed with formaldehyde (Thermo Scientific, Rockford, IL, USA), rinsed twice with 0.13 M 1-methylimidazole, and finally fixed again with 1-ethyl-3-(3-dimethylaminopropyl) carbodiimide (EDC, Thermo Scientific). After blocking endogenous peroxidase in H_2_O_2_, prehybridization buffer was added, and the slides were finally hybridized with 200 nM digoxigenin (DIG)-labeled, locked nucleic acid (LNA)-modified oligonucleotides.

Two senior pathologists blinded to the clinical data scored the tissue microarray staining independently using the following criteria. Intensity of immunostaining was scored as 0 (no immunostaining), 1 (weak immunostaining), 2 (moderate immunostaining), and 3 (strong immunostaining). The percentage of immunoreactive cells was documented as 0 (none), 1 (<20%), 2 (20–50%), 3 (51–75%), and 4 (>75%). Cutoff values for low and high expression groups were determined by using the degree × intensity staining rank. Low expression was defined as a final score < 6 and high expression was defined as a final score ≥ 6.

### Cell culture

Human ESCC cell lines (TE-1 and KYSE510) were purchased from American Type Culture Collection (Manassas, VA, USA). All cells were prepared in 10% fetal bovine serum (FBS; Gibco, Grand Island, NY, USA) in Roswell Park Memorial Institute (RPMI) 1640 medium (Gibco). Culture dishes containing cells were incubated at 37 °C in humidified air with 5% CO_2_.

### Total RNA extraction and quantitative real-time reverse transcription PCR (qRT-PCR)

Total RNA was extracted from frozen tissues and cells using RNAiso Plus (Takara, Shiga, Japan) according to the manufacturer’s instructions. cDNA was synthesized from the total RNA using PrimeScript RT Master Mix (Takara, Dalian, China). The RNA was quantified using the cDNA as the template for qPCR with the SsoFast EvaGreen Supermix (Bio-Rad Laboratories, Hercules, CA, USA), and the *GAPDH* gene (encoding glyceraldehyde-3-phosphate dehydrogenase) was used as an internal reference. qPCR was performed with the following thermal cycling program: 95 °C for 30 s; 40 cycles at 95 °C for 5 s, 60 °C for 30 s; and a final dissociation step. All primer sequences use are shown in Table S2.

### Cell transfection

CircRNA overexpression and short hairpin RNA (shRNA) lentiviral vectors, miRNA lentiviral vectors, and matching negative control vectors were designed and synthesized by Synbio Technologies (Suzhou, China). Small interfering RNA (siRNA) sequences were directly synthesized (GenePharma, Shanghai, China). Lipofectamine 3000 (Invitrogen, Carlsbad, CA, USA) was used to transfect cells with the designated lentiviral vectors according to the manufacturer’s instructions. All shRNA sequences are listed in Table S3A.

### Fluorescence in situ hybridization (FISH)

The FISH probes were designed and provided by RiboBio (Guangzhou, China). Cyanine (Cy3)-labeled probes and Fluorescein amidite (FAM)-labeled probes were used as markers for hsa_circ_0021727 and miRNA-23b-5p, respectively (Table S2B). Nuclei were stained with 4′,6-dimethyl-2-phenylindole (DAPI). The assay was performed according to the instructions of the FISH kit (Gene Pharma, Shanghai, China). Stained images were acquired using confocal microscopy (Leica, Wetzlar, Germany).

### Cell proliferation, colony formation assays, and 5-Ethynyl-2′-deoxyuridine (EdU) incorporation assay

[4,5-Dimethylthiazol-2-yl]-2,5-diphenyltetrazolium bromide (MTT) determination was used to assess cell proliferation. The transfected cells were seeded in a 96-well plate, then 20 µL of 5 mg/mL MTT solution (MTT Cell Proliferation and Cytotoxicity Assay Kit, BOSTER, Wuhan, China) was added and incubated for 4 h. Finally, 100 µL of dimethyl sulfoxide was added. A microplate reader was then used to measure the optical density at 490 nm.

For the colony formation assays, transfected cells were divided into 6-well plates (1000 cells/well). After 2 weeks in culture, the cells were fixed with 75% ethanol and stained with 0.2% crystal violet. The colony formation rate was determined by counting the number of stained colonies.

The EdU immunofluorescence assay was performed using a Cell-Light EdU DNA Cell Proliferation Kit (RiboBio) according to the manufacturer’s instructions. Treated ESCC cells were incubated with EdU for 3 h. After fixation and permeabilization, anti-EdU reagents and DAPI were used for cell staining. Fluorescence microscopy was used to observe and obtain images.

### Cell invasion, migration, and wound healing assay

For the invasion assay, medium was added to the lower chamber of the Transwell apparatus after precoating the upper chamber membrane with 100 μL of Matrigel (BD Bioscience, San Jose, CA, USA). Then, 100 μL of serum-free medium was added to the transfected cells, which were added to the upper chamber, complete medium was added to the lower chamber, and incubated at 37 °C in 5% CO_2_ for 24 h. Finally, the cells were fixed with 4% paraformaldehyde solution and stained with 0.1% crystal violet solution. Images were obtained using an inverted fluorescence microscope using Olin and the cells were counted.

Three-dimensional (3D) spheroid invasion assays were used to detect the invasive ability of tumor cells. Transfected cells were seeded in ultra-low attachment (ULA) round-bottom 24-well plates for 4 days, allowing them to form tumor spheroids. Then, 100 µL of basement membrane matrix (BMM, Corning Inc., Corning, NY, USA) was added to each well, followed by centrifugation and incubation at 37 °C for 1 h to solidify. Finally, 100 µL of medium containing 10% FBS was added to each well and the plates were incubated at 37 °C in 5% CO_2_. We obtained images under an inverted microscope.

For the wound healing assay, transfected cells were added to a six-well plate culture in serum-free medium, incubated to form a confluent cell monolayer, followed by linear wounding using a pipette tip. Representative images of the wound were captured at 0 h and 24 h after wound formation under a microscope. The difference in the width of the wound between 0 h and 24 h was used as a measure of cell migration.

### Western blotting analysis

We used Radioimmunoprecipitation assay (RIPA) buffer to the lyse transfected cells, obtained the supernatant, and added protease inhibitors (1%; ComWin Biotech, Beijing, China). The protein concentration was assessed using a bicinchoninic acid (BCA) Kit (Beyotime, Jiangsu, China). Equal amounts of protein were separated using sodium dodecyl sulfate-polyacrylamide gel electrophoresis (SDS-PAGE) (10% or 8%) and transferred to 0.45 µm polyvinylidene fluoride (PVDF) membranes (Roche). After blocking with 5% nonfat milk for 1 h, the PVDF membranes were incubated with primary antibodies overnight at 4 °C. Membranes were washed a minimum of three times in Tris-buffered saline-Tween 20 (TBST) for 10 min each time, followed by 1 h of incubation with goat anti-rabbit or goat anti-mouse secondary antibodies. Finally, the membrane was washed three times with TBST again. The immunoreactive protein bands were detected using SuperSignal West Femto Agent (Millipore, Billerica, MA, USA) and visualized using the Chemical Mp Imaging System (Bio-Rad). The antibodies used are listed in Table S4.

### RNA pull-down assay

An hsa_circ_0021727 biotin-conjugated probe and oligo probe (Table S3B) were designed by RiboBio. Lysates of treated cells were incubated with streptavidin magnetic beads (Life Technologies, Carlsbad, CA, USA) for 2 h. The cell lysates were then incubated with probe-coated beads at 4 °C overnight. The beads were washed five times repeatedly, and then the bound miRNAs in the pulled down material were extracted using the Trizol reagent and analyzed using qRT-PCR analysis.

### Luciferase reporter assay

The miR-23b-5p sequence and the miR-23b-5p-mut sequence were designed to synthesize luciferase reporter plasmids. The luciferase reporter plasmids (pGL3-miR-23b-5p, pGL3-miR-23b-5p-mut, and pGL3-basic-vector) were synthesized by Generay Biotech (Shanghai, China). The recombinant luciferase reporter plasmids were inserted with the 3′ untranslated region (UTR) of *TAB1* (encoding TAK1-binding protein 10). PCR primers sequences are shown in Table S2. When the ESCC cells grew to 80% confluence, they were co-transfected with other lentiviral vectors, luciferase reporter plasmids, and pGL3-basic vector. Cell lysates were sampled after 48 h, and luciferase activity was analyzed using a dual-luciferase reporter gene assay system (Promega, Madison, WI, USA).

### RNA-seq assay

Total RNA of ESCC cells was extracted in accordance with the manual of the TRIzol® reagent (Invitrogen, Shanghai, China).VAHTS Stranded mRNA-seq Library Prep Kit for Illumina V2 (Vazyme Biotech, NR612-02) was used for library preparation according to the instructions.Reads were aligned to the human Ensemble genome GRCh38 (mouse Ensemble genome GRCm38) using Hisat2 aligner (v2.1.0) under parameters:“--rna-strandness RF”.The reads mapped the genome were calculated using featureCounts (v1.6.3).Differential gene expression analysis was performed using the DESeq2 R.

### Animal experiments

Male BALB/c nude mice (20 ± 2 g) were purchased from Guangdong Medical Laboratory Animal Center (MLAC). All animal experiments were performed in accordance with the principles and procedures outlined in the Gannan Medical University Guide for the Care. Approval was obtained from the First Affiliated Hospital of Gannan Medical University Animal Ethics Committee.

To observe tumor growth, transfected cells were injected subcutaneously into the left side of the axilla. Tumor growth was evaluated by detecting the volumes of the xenografts once each week. Specifically, the formula for calculating the volume was: (volume) = 1/2 × (long axis) × (short axis).

To establish a lung metastasis model, cancer cells stably expressing firefly luciferase were injected into 4-week-old BALB/c nude mice via the tail vein. Six weeks after injection, bioluminescent pictures of tumor lung metastases were obtained using an in vivo imaging system. The mice were sacrificed, and their lung tissue was excised to record the number of lung metastases; histological sections and HE staining were performed.

### Statistical analysis

SPSS (version 22.0, IBM Corp., Armonk, NY, USA) and GraphPad Prism 9.1 (GraphPad Software Inc., La Jolla, CA, USA) were used for statistical analysis. Student’s *t*-test and/or chi-squared test were employed for data analysis. The results were indicated as mean ± SD. *P* < 0.05 was considered significant.

## Results

### Expression and identification of hsa_circ_0021727 in ESCC tissues and cells

**Fig. 1:**
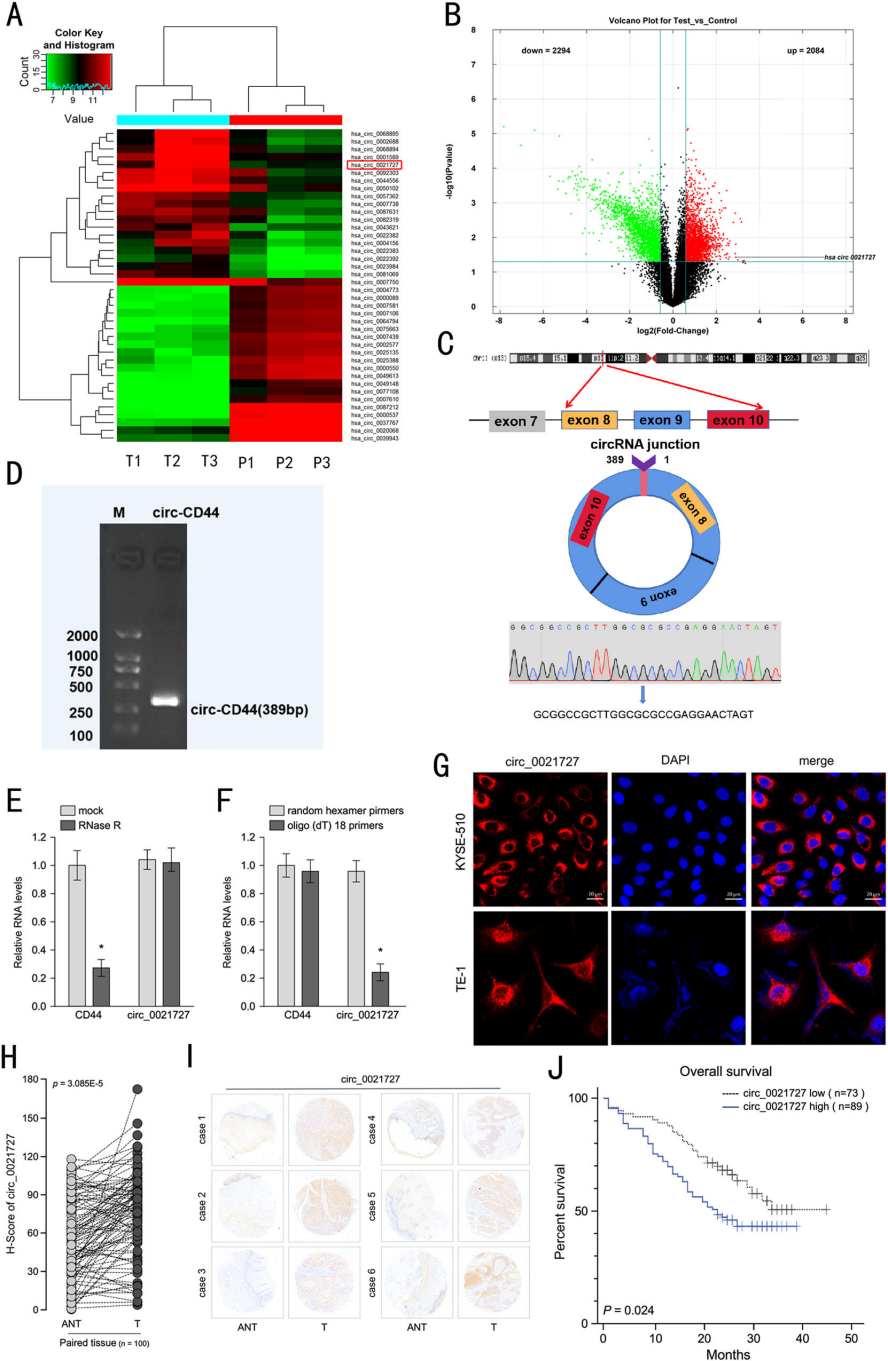
Expression and identification of hsa_circ_0021727. **A** Heat maps of differentially expressed circRNAs in three ESCC tissues (T) and adjacent normal tissues (P). **B** Volcano plots showing changes in circRNA expression between cancerous and paracancerous tissues. **C** Schematic and Sanger sequencing of hsa_circ_0021727. **D** Agarose gel electrophoresis detection of hsa_circ_0021727 in KYSE510 cells. **E** The relative RNA levels were examined by qRT-PCR after treatment with RNase R or mock among total RNAs derived from KYSE 510 cells. **F** Random hexamer or oligo (dT)18 primers were utilized for reverse transcription assays. The relative RNA levels were examined using qRT-qPCR and normalized to those generated using random hexamer primers. **G** The subcellular localization of hsa_circ_0021727 in KYSE510 and TE-1 cells performed using FISH. **H** and **I** RNA-ISH detection of the expression of hsa_circ_0021727 in ESCC tissues and adjacent tissues. **J** Kaplan–Meier analysis of the OS in 162 patients with ESCC with high or low expression of hsa_circ_0021727. Values are expressed as the means ± SD; *P < 0.05.

To explore the expression of circRNAs in ESCC tissues and paracancerous tissues, three pairs of samples (three ESCC tissues and three paracancerous tissues) were used for circRNA microarray analysis. We found that 7518 circRNAs were expressed in ESCC tissues and paracancerous tissues. Twenty upregulated or downregulated circRNAs were screened, among which hsa_circ_0021727 was the most upregulated circRNA in cancer tissues compared with normal tissues (Fig. 1A). Volcano plots showed the changes in circRNAs expression between cancerous and paracancerous tissues (Fig. 1B). Hsa_circ_0021727 is derived from the *CD44* gene (encoding CD44 molecule (Indian blood group)) and is spliced from exon 8 to exon 10. Sanger sequencing confirmed the junction sequence in the divergent primers spanning the predicted products (Fig. 1C). Agarose gel electrophoresis also verified the existence of hsa_circ_0021727 (Fig. 1D).We detected the expression of hsa_circ_0021727 in ET-1A, TE-1 and KYSE510 cells by qRT-PCR(Fig. S1). The results showed thathsa_ circ_ 0021727 was upregulated in ESCC cells. We then confirmed the cyclic character of hsa_circ_0021727. First, we digested total RNA using RNase R. The results showed that hsa_circ_0021727 had stronger RNase R resistance than linear *CD44* (Fig. 1E). Then, we designed random hexamer or oligo(dT)18 primers for qPCR. The relative amplification of hsa_circ_0021727 was obviously lower when oligo (dT)18 primers were applied than when random hexamer primers were used, while the amplification of linear *CD44* was unchanged (Fig. 1F). FISH results showed that hsa_circ_0021727 was mainly located in the cytoplasm (Fig. 1G). The clinicopathological data of the patients in the tissue microarrays are provided in Table S1A. RNA-ISH revealed that the expression of hsa_circ_0021727 was higher in cancer tissues of patients with ESCC than in adjacent tissues (Fig. 1H, I). 54.9% of the patients had high expression of hsa_circ_0021727 (Table S1B). The expression of hsa_circ_0021727 was not statistically significantly associated with the patient’s age, clinical analysis, distant metastasis, and lymph node metastasis, but was significantly correlated with patient survival according to Cox multivariate analysis (Table 1). Thus, the expression of hsa_circ_0021727 can be used as an independent prognostic factor for patients (Table 2). The survival time of patients with high expression of hsa_circ_0021727 was significantly lower than that of patients with low expression of hsa_circ_0021727 (Fig. 1J).

**Table 1:** Correlation between circ_0021727 expression and clinicopathological characteristics of esophageal cancer.

Characteristics		circ_0021727		Chi-square test *P*-value	Fisher’s exact test *P*-value
		Low No. cases	High No. cases		
Age (years)	>55	63	69	0.109	0.109
	≤55	10	20		
AJCC clinical stage	I~II	40	39	0.109	0.109
	III~IV	33	50		
T classification	T1~T2	11	17	0.322	0.322
	T3~T4	62	72		
N classification	N0	40	38	0.084	0.084
	N1~N3	33	51		
M classification	No	73	87	0.300	0.300
	Yes	0	2		
Gender	Male	63	77	0.573	0.573
	Female	10	12		
Survive or Mortality	Survive	44	39	0.027	0.027
	Mortality	29	50

**Table 2:** Univariate and multivariate analyses of various prognotic parameters in patients with esophageal cancer by cox-regression analysis.

	Univariate analysis			Multivariate analysis		
	No. patients	*P*	Relative risk	*P*	Relative risk	95% confidence interval
M stage						
M0	160	0.001	10.982	0.012	6.841	1.538-30.430
M1	2					
AJCC stage						
I~II	79	0.002	2.102	0.003	2.035	1.277-3.243
III~IV	83					
Expression of circ00212727						
Low expression	73	0.027	1.679	0.041	1.620	1.021-2.572
High expression	89					

### Hsa_circ_0021727 promotes migration, invasion, and proliferation of ESCC cells in vitro

**Fig. 2:**
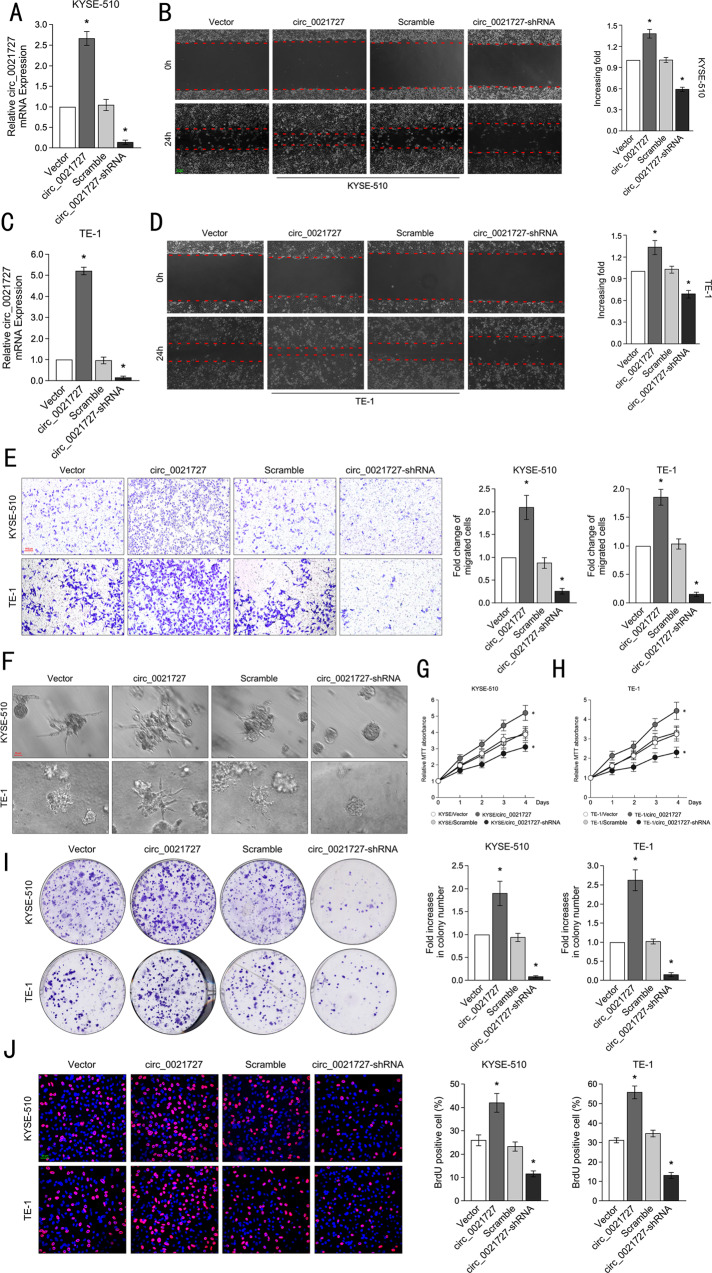
Hsa_circ_0021727 promotes the migration, invasion, and proliferation of ESCC cells in vitro. **A** and **C** qRT-PCR detection of the expression efficiency of hsa_circ_0021727 after cell transfection. **B** and **D** Wound healing assays for the migration ability of KYSE510 and TE-1 cells after cell transfection. **E** Transwell assays examining the invasive ability of KYSE510 and TE-1 cells after transfection of the hsa_circ_0021727 overexpression or knockdown vectors. **F** Three-dimensional (3D) spheroid invasion assays showing the effects of hsa_circ_0021727 on the growing antennae of KYSE510 and TE-1 cells. **G** and **H** MTT assays detecting the proliferation of KYSE510 and TE-1 cells after transfection of hsa_circ_0021727 overexpression or knockdown vectors. **I** Colony formation assays examining the proliferative capacity of KYSE510 and TE-1 cells transfected with hsa_circ_0021727 overexpression or knockdown lentiviral vectors. **J** EdU immunofluorescence assays detecting the proliferative capacity of KYSE510 and TE-1 cells. Values are expressed as the means ± SD; *P < 0.05.

To understand the function of hsa_circ_0021727, we designed overexpression and knockdown lentiviral vectors for hsa_circ_0021727. qRT-PCR confirmed the efficiency of lentiviral vector transfection (Fig. 2A, C). The migration ability of ESCC cells was detected by wound healing assays. The results suggested that the migration ability of ESCC cells was enhanced after overexpression of hsa_circ_0021727. In contrast, knockdown of hsa_circ_0021727 inhibited the migration ability of ESCC cells (Fig. 2B, D). The results of Transwell assays showed that hsa_circ_0021727 promoted the invasion ability of ESCC cells, while hsa_circ_0021727 deficiency inhibited the invasion ability of ESCC cells (Fig. 2E). Furthermore, in 3D spheroid invasion assays, ESCC cells overexpressing hsa_circ_0021727 had more cellular antennae than control cells and displayed morphological changes typical of highly invasive cells. However, the results in the hsa_circ_0021727 knockdown group were the opposite (Fig. 2F). The results of MTT assays revealed that cells transfected with the hsa_circ_0021727 overexpressing vector had a stronger proliferation ability. After knockdown of hsa_circ_0021727, the proliferation ability of ESCC cells was significantly inhibited (Fig. 2G, H). We also verified the proliferation of ESCC cells by colony clone formation assays, which showed that overexpression of hsa_circ_0021727 promoted cell proliferation, while knockdown of hsa_circ_0021727 inhibited cell proliferation (Fig. 2I). EdU immunofluorescence assays showed the same results (Fig. 2J). Taken together, these results suggested that hsa_circ_0021727 promoted ESCC cell proliferation, invasion, and migration.

### Hsa_circ_0021727 promotes metastasis and proliferation of ESCC cells in vivo

**Fig. 3:**
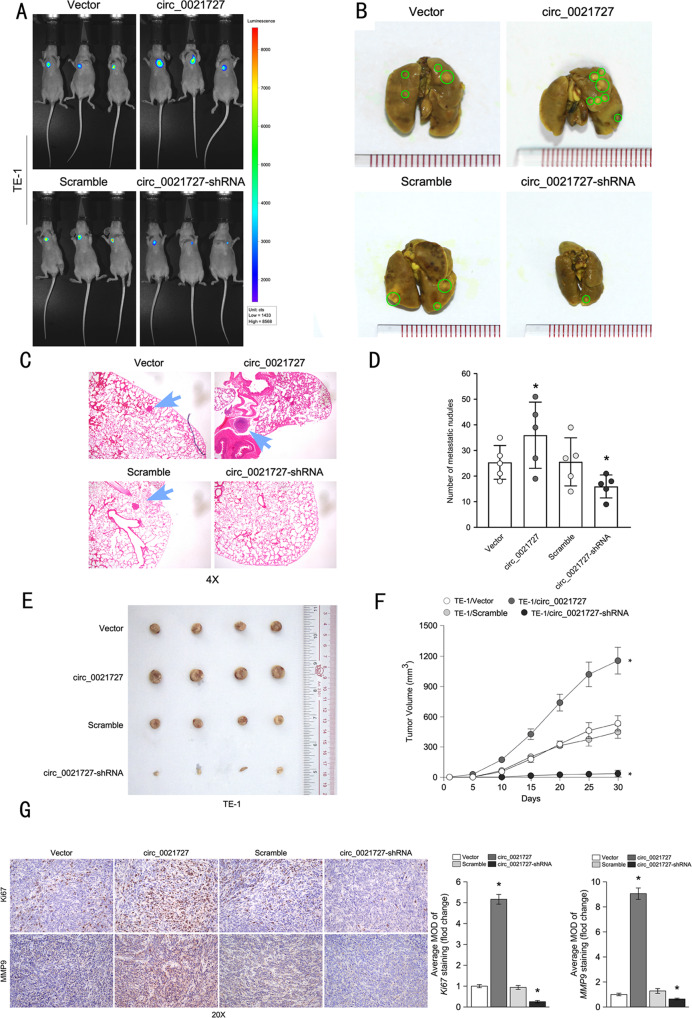
Hsa_circ_0021727 promotes the metastasis and proliferation of ESCC cells in vivo. **A** Representative images and analysis of luminescence intensity in tail vein tumor metastasis mouse models. **B** Images of lung tissue in tail vein tumor metastasis mouse models. **C** Lung tissue was stained with HE. **D** Statistical analysis of lung metastatic nodules. **E** Representative image of a subcutaneous xenograft tumor. **F** Growth curves of tumor volumes in subcutaneous xenograft tumors models. **G** Immunohistochemistry showing the expression of Ki-67 and MMP9 in mouse tumor tissues (values are expressed as the means ± SD; *P < 0.05).

To evaluate the effect of hsa_circ_0021727 on proliferation and metastasis in vivo, we performed subcutaneous tumorigenesis and mice tail vein injection assays in BALB/c nude mice. Stably transfected hsa_circ_0021727 overexpressing or knockdown ESCC cells (TE-1) were used for the experiments. For the lung metastasis model, we first observed intravital fluorescence imaging, and then sacrificed the mice and examined the formation of metastatic tumor nodules in the lungs. The mice injected with hsa_circ_0021727 overexpressing cells had more metastatic nodules than the mice injected with hsa_circ_0021727 knockdown cells (Fig. 3A–D). Subcutaneously injected BALB/c nude mice were used to assess tumor growth. The results showed that the tumor volume and weight of the hsa_circ_0021727 overexpression group increased significantly compared with that of the empty vector group. At the same time, the volume and weight of tumors in the hsa_circ_0021727 knockdown group were significantly reduced compared to those of the scramble group (Fig. 3E, F). We also verified the expression of hsa_circ_0021727 in mouse tumor tissues by qRT-PCR(Fig. S3A). Immunohistochemical analysis showed that the expression of MKI67 (marker of proliferation Ki-67) and matrix metalloproteinase 9 (MMP9) in mouse tumors correlated positively with the expression of hsa_circ_0021727(Fig. 3G).

### Hsa_circ_0021727 functions as a sponge for miR-23b-5p

**Fig. 4:**
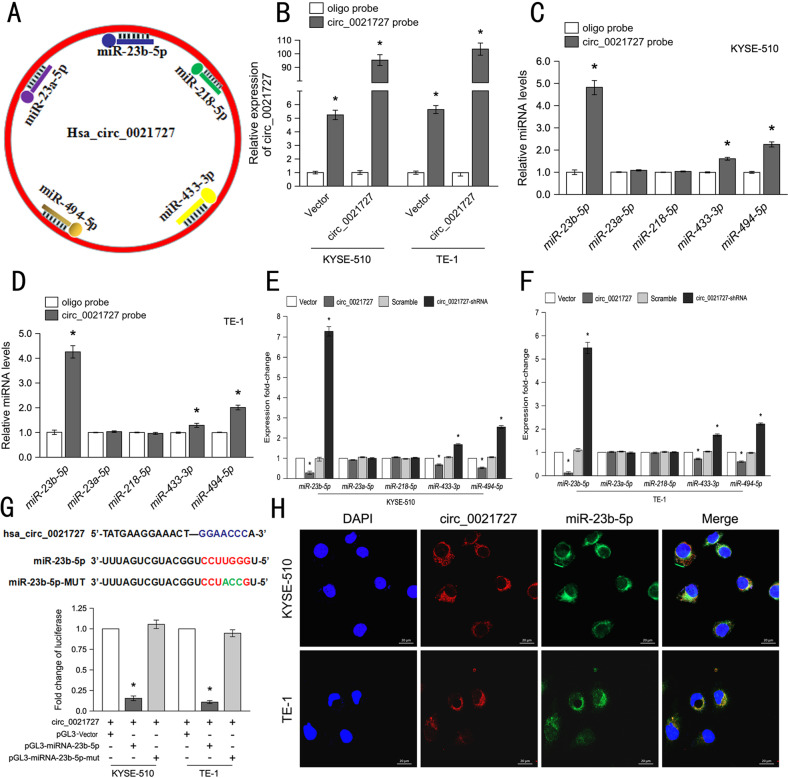
Hsa_circ_0021727 functions as a sponge for miR-23b-5p. **A** Image suggesting that hsa_circ_0021727 might target bound miRNAs. **B** Probe efficiency for qRT-PCR detection. **C** and **D** Relative levels of five miRNAs in KYSE510 and TE-1 cells lysates pulled down by the hsa_circ_0021727 probe or the oligo probe. **E** and **F** qRT-PCR detection of the levels of five miRNAs in KYSE510 and TE-1 cells transfected with hsa_circ_0021727 overexpression or knockdown lentiviral vectors. **G** A luciferase reporter gene assay performed in KYSE510 and TE-1 cells co-transfected with hsa_circ_0021727 overexpression vector, pGL3 plasmid, pGL3-miR-23b-5p-wt, and pGL3-miR-23b-5p-mut. **H** FISH analysis showing the colocalization between hsa_circ_0021727 and miR-23b-5p in KYSE510 and TE-1 cells. Values are expressed as the means ± SD; *P < 0.05.

CircRNAs acting as sponges for miRNAs have been well reported. To search for miRNAs that might bind to hsa_circ_0021727, we selected five miRNAs (miR-23a-5p, miR-23b-5p, miR-218-5p, miR-433-3p, and miR-494-5p) with the highest scores in the miRanda database (Fig. 4A). By using TCGA dataset, we found no statistical difference in the expression of miR-23b-5p between ESCC and normal tissues(Fig. S2B). However, esophageal cancer patients with high expression of miR-23b-5p had a longer survival period than esophageal cancer patients with low expression of miR-23b-5p(Fig. S2C). We designed a biotinylated hsa_circ_0021727 probe and an oligonucleotide probe. We then verified the pull-down efficiency of the hsa_circ_0021727 probe (Fig. 4B). The RNA pull-down results revealed that among the five candidate miRNAs, only miR-23b-5p could be markedly pulled down by the hsa_circ_0021727 probe in ESCC cells transfected with the hsa_circ_0021727 overexpressing vector (Fig. 4C, D). The qRT-PCR results also confirmed that the expression of hsa_circ_0021727 correlated negatively with the expression of miR-23b-5p (Fig. 4E, F).We detected the expression of miR-23b-5p in tumor tissues of mice(Fig. S3B). The expression of hsa_circ_0021727 correlated negatively with the expression of miR-23b-5p. To further verify that hsa_circ_0021727 can bind to miR-23b-5p, we first designed a mutant of miR-23b-5p, and then performed luciferase reporter gene analysis. The results showed that hsa_circ_0021727 attenuated the luciferase activity of the pLG3-miR-23b-5p-WT (wild-type) reporter, but could not attenuate the luciferase activity of the pLG3-miR-23b-5p-mutreporter (Fig. 4G). Furthermore, FISH analysis found that hsa_circ_0021727 and miR-23b-5p co-localized in the cytoplasm (Fig. 4H). Overall, these results indicated that hsa_circ_0021727 acts as a sponge for miR-23b-5p.

### Hsa_circ_0021727 promotes ESCC cell proliferation, invasion, and migration via miR-23b-5p

**Fig. 5:**
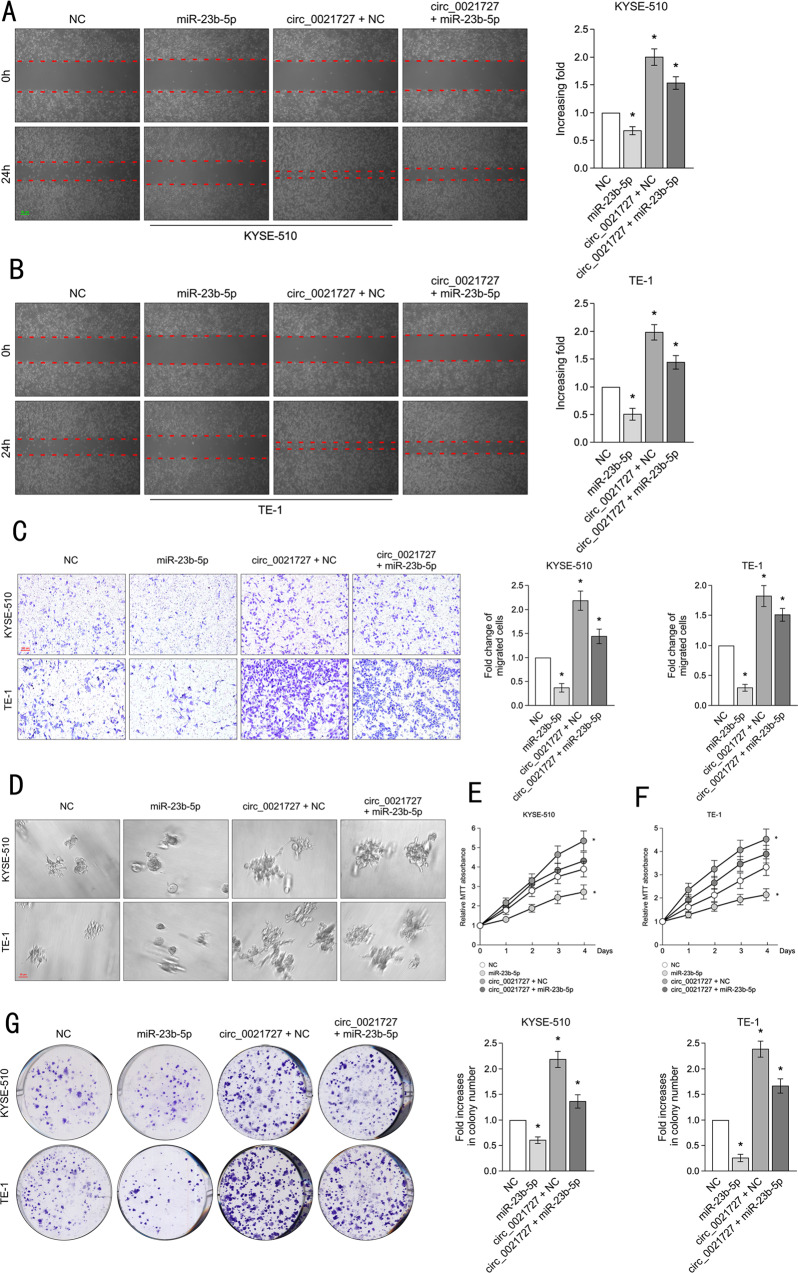
Hsa_circ_0021727 promotes ESCC cell proliferation, invasion, and migration via miR-23b-5p. **A** and **B** Wound healing assays evaluating the cell migration ability of KYSE510 cells and TE-1 cells co-transfected with miR-23b-5p and hsa_circ_0021727 overexpression vectors. **C** Transwell assays showing that miR-23b-5p and hsa_circ_0021727 jointly affected the invasion ability of KYSE510 and TE-1 cells. **D** Three-dimensional (3D) spheroid invasion assays showing the effects of hsa_circ_0021727 and miR-23b-5p on the growing antennae of KYSE510 and TE-1 cells. **E** and **F** MTT assays demonstrating the proliferation capacity of KYSE510 and TE-1 cells co-transfected with miR-23b-5p and hsa_circ_0021727 overexpression vectors. **G** Colony formation assays indicating that the cell proliferation capability of KYSE510 and TE-1 cells transfected with miR-23b-5p was reversed when the cells were co-transfected with hsa_circ_0021727 (values are expressed as the means ± SD; *P < 0.05).

To investigate the effect of miR-23b-5p on the function of ESCC cells, the miR-23b-5p overexpression vector, the control vector for miR-23b-5p overexpression (NC), or the hsa_circ_0021727 overexpression vector were co-transfected into ESCC cells.We detected the transfection efficiency of the hsa_circ_0021727 and miR-23b-5p in ESCC cells by qRT-PCR(Fig. S4A–D). The wound healing assay showed that miR-23b-5p overexpression increased the width of the linear wounds. After co-transfection of hsa_circ_0021727 and miR-23b-5p overexpression vectors, miR-23b-5p reduced the migration ability of ESCC cells (Fig. 5A, B). The results of the Transwell assays showed that the invasion ability of ESCC cells was weakened after overexpression miR-23b-5p. After co-transfection of hsa_circ_0021727 overexpression vector, miR-23b-5p neutralized the effect of hsa_circ_002172 (Fig. 5C). This indicated that hsa_circ_0021727 was a negative regulator of miR-23b-5p. We observed the same trend in the 3D spheroid invasion assays (Fig. 5D). The results of the MTT assays indicated that the proliferation capacity of the cells was attenuated after transfection of miR-23b-5p alone. After co-transfection of hsa_circ_0021727 and miR-23b-5p overexpression vectors, miR-23b-5p attenuated the effect of hsa_circ_0021727 (Fig. 5E, F). Colony formation assays confirmed these results (Fig. 5G). Our results confirmed that hsa_circ_0021727 could promote ESCC cell proliferation, invasion, and migration by negatively regulating miR-23b-5p.

### Hsa_circ_0021727 promotes the metastasis and proliferation of ESCC cells in vivo by regulating the expression of miR-23b-5p

**Fig. 6:**
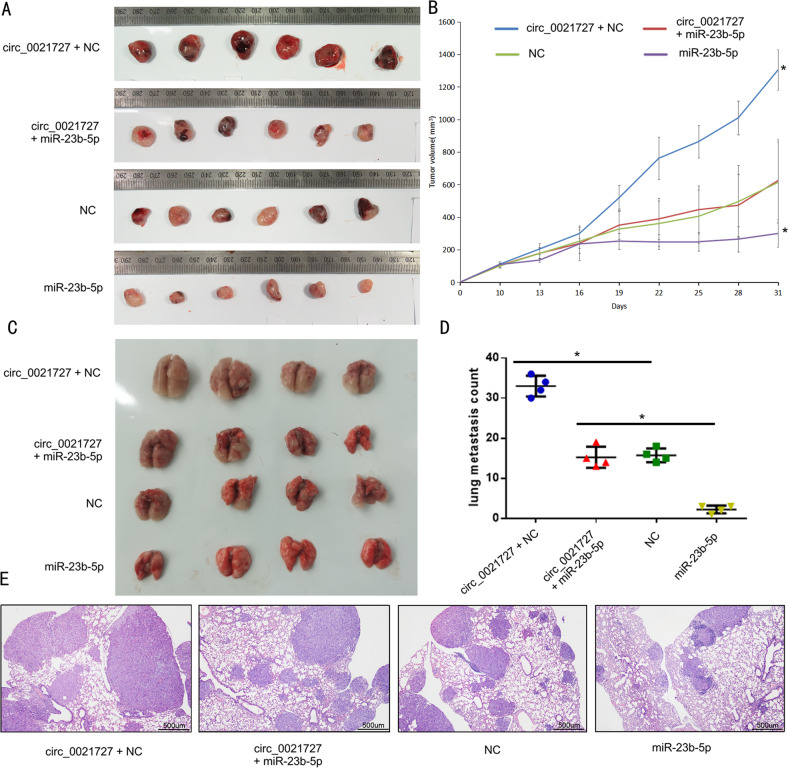
Hsa_circ_0021727 promotes the metastasis and proliferation of ESCC cells in vivo by regulating the expression of miR-23b-5p. **A** Image showing the subcutaneous xenograft tumor tissues injected with KYSE510 cells co-transfected with miR-23b-5p and hsa_circ_0021727 overexpression vectors. **B** Graphs of tumor volumes in subcutaneous xenograft tumor models. **C** Image of lung metastatic nodules. Nude mice were injected with KYSE510 cells co-transfected with miR-23b-5p and hsa_circ_0021727 overexpression vectors. **D** Statistical analysis of lung metastatic nodules. **E** Lung tissue was stained using HE. Values are expressed as the means ± SD; *P < 0.05.

The miR-23b-5p overexpression vector, the control vector for miR-23b-5p overexpression (NC), or the hsa_circ_0021727 overexpression vector were used to co-transfect KYSE510 cells. They were divided into four groups, namely the miR-23b-5p overexpression group, the NC group, the circ_0021727+miR-23b-5p overexpression group, and the circ_0021727+NC group. The treated ESCC cells were subcutaneously inoculated into BALB/c nude mice. It was found that miR-23b-5p could inhibit tumor growth in vivo. Hsa_circ_0021727 attenuated the effect of miR-23b-5p in vivo (Fig. 6A, B).We detected the expression of hsa_circ_0021727, miR-23b-5p and TAB1 in mouse tumor tissues. The mRNA expression of miR-23b-5p was negatively regulated by hsa_circ_0021727, while miR-23b-5p did not affect the expression of hsa_circ_0021727(Fig. S4E, F). The protein expression of TAB1 was positively correlated with the expression of hsa_circ_0021727,and negatively correlated with the expression of miR-23b-5p (Figs. S3C and S4G). The four groups of cells were injected into the tail vein of BALB/c nude mice. The results of the lung metastasis model showed that transfection of miR-23b-5p resulted in fewer metastatic nodules in mice. However, after co-transfection of hsa_circ_0021727, metastatic pulmonary nodules were significantly increased (Fig. 6C, D). We also sectioned and hematoxylin and eosin (HE) stained the metastatic nodules. The results showed that the group transfected with the hsa_circ_0021727 overexpressing vector had more metastatic nodules, while the group transfected with the miR-23b-5p overexpressing vector had fewer metastatic nodules (Fig. 6E). Therefore, we believed that hsa_circ_0021727 promoted the metastasis and proliferation of ESCC cells in vivo via downregulating miR-23b-5p.

### Hsa_circ_0021727 activates TAB1/NFκB signaling via miR-23b-5p

**Fig. 7:**
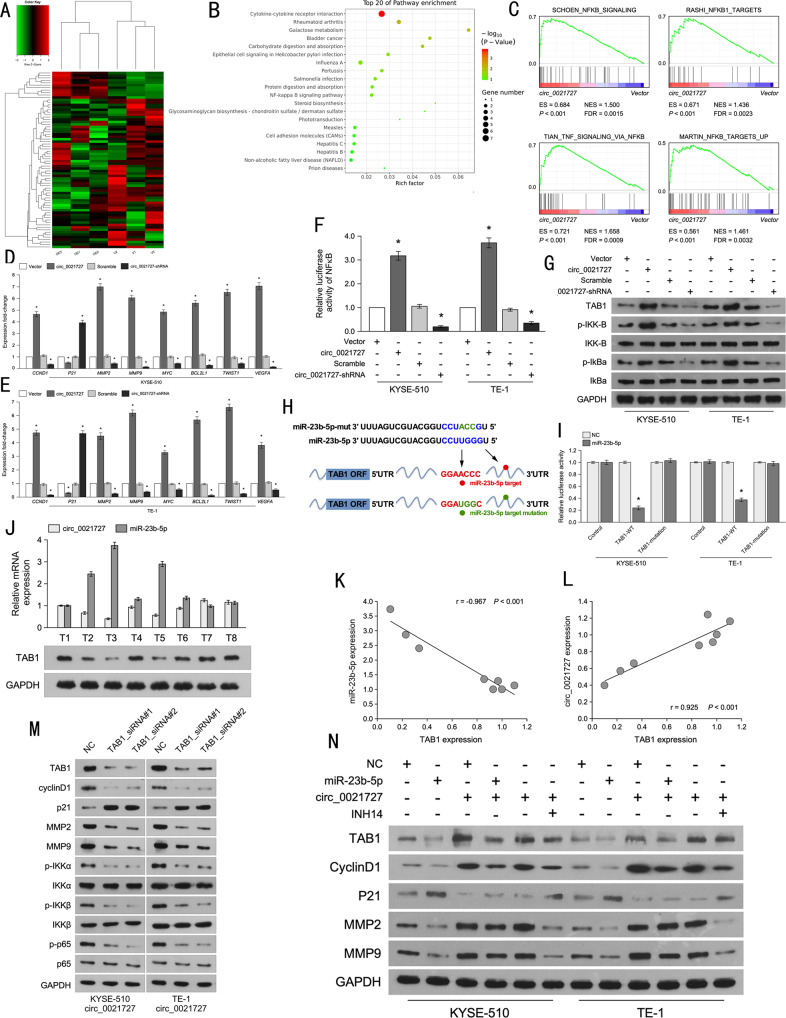
Hsa_circ_0021727 activates TAB1/NFκB signaling via miR-23b-5p. **A** Heatmap created from mRNA-seq (OE: overexpression, V: vector). **B** The top 20 enriched pathways in the mRNA data. **C** GSEA results showing that hsa_circ_0021727 correlated positively with NFκB signaling. **D** and **E** qRT-PCR detection of the expression of NFκB signaling-related factors in cells overexpressing or knocked down for hsa_circ_0021727. **F** Dual-luciferase assay detecting the luciferase activity related to NFκB signaling in cells overexpressing or knocked down for hsa_circ_0021727. **G** In cells overexpressing or knocked down for hsa_circ_0021727, western blotting was used to detect the protein levels of TAB1 and NFκB pathway factors. **H** TAB1 mutant and wild-type sequences were designed. **I** Dual-luciferase assays showing the luciferase activity after co-transfection of miR-23b-5p overexpression vector or NC vector, TAB1 wild-type or TAB1 mutant vector. **J** qRT-PCR and western blotting analyses of the expression of hsa_circ_0021727, miR-23b-5p, and TAB1 in ESCC patient tissues. **K** Analysis of the correlation between miR-23b-5p and TAB1. **L** Analysis of the correlation between hsa_circ_0021727 and TAB1. **M** In cells overexpressing hsa_circ_0021727 and knocked down for *TAB1*, western blotting examined the protein level of TAB1 and downstream related factors. **N** In cells overexpressing hsa_circ_0021727 and miR-23b-5p, western blotting examined the protein level of TAB1 and downstream related factors. Western blot detection of the protein level of TAB1 and downstream related factors in KYSE510 and TE-1 cells overexpressing hsa_circ_0021727 and treated with INH14. Values are expressed as the means ± SD; *P < 0.05.

To determine the downstream pathways regulated by hsa_circ_0021727, we performed mRNA-seq using ESCC cells (KYSE510) transfected with the hsa_circ_0021727 overexpression vector and empty vector. Heat maps revealed the expression of downstream genes (Fig. 7A). The top 20 GO terms for biological process were screened out (Fig. 7B). The results of Gene Set Enrichment Analysis (GSEA) showed that the expression of hsa_circ_0021727 correlated significantly and positively with NFκB signaling (Fig. 7C). Expression difference of NFκB signaling-related genes(mRNA-seq) included in Fig. S5. The mRNA expression of TAB1(mRNA-seq) included in Fig. S6.qRT-PCR verified that the expression of hsa_circ_0021727 was highly correlated with the expression of downstream factors of the NFκB pathway (Fig. 7D, E). We showed that hsa_circ_0021727 acts as a sponge for miR-23b-5p. Therefore, to identify possible target genes of miR-23b-5p in ESCC cells, we used online software (miRanda) to make predictions. We found that miR-23b-5p had a binding site for TAB1. TAB1 is an adapter protein constitutively associated with N-terminal kinase domain of TGF-beta activated kinase 1 (TAK1) even in unstimulated cells, while TAB2 and TAB3 bind to the C-terminus of TAK1 through their TAK1-binding domains after stimulation [29, 30]. TAB1, TAB2, TAB3, and TAK1 form the TAK1-TABs complex [31]. The TAK1-TABs complex phosphorylates inhibitor of nuclear factor kappa B kinase subunit beta (IKKβ) at Ser177 and Ser181, which is required for the activation of NF-κB signaling [32, 33]. Through dual-luciferase experiments, we found that hsa_circ_0021727 could enhance the luciferase activity of NF-κB (Fig. 7F). Western blotting analysis showed that the protein levels of TBA1, phosphorylated (p)-IKKβ and p-NF-κB inhibitor alpha (IKBα) were upregulated after overexpression of hsa_circ_0021727. By contrast, the levels of TBA1, p-IKKβ, and p-IKBα were downregulated after transfection of circ_0021727-shRNA (Fig. 7G). We performed luciferase reporter assays by constructing wild-type (WT) and mutant type (MUT) pGL3-Basic reporter vector for *TAB1* (Fig. 7H). Compared with the negative control (NC), miR-23b-5p significantly reduced the luciferase activity of the *TAB1*-WT reporter, while miR-23b-5p did not affect the luciferase activity of the *TAB1*-MUT reporter (Fig. 7I). Therefore, we believe that miR-23b-5p could target and bind to *TAB1*. To explore the relationship between hsa_circ_0021727, miR-23b-5p, and TAB1, we selected eight specimens from patients with ESCC for qRT-PCR and western blotting analysis. The results revealed that hsa_circ_0021727 expression correlated negatively with the expression of miR-23b-5p, but positively with the expression of TAB1 (Fig. 7J–L). In TCGA dataset, there was no statistical difference(*p* = *0.239*) between the expression of TAB1 and the survival time of ESCC patients(Fig. S2C). N-(4-Ethylphenyl)-N′-phenylurea (INH14) reduced NF-kB activation by inhibiting IKK [34], thus we could block the NFκB pathway by adding INH14. Through western blotting analysis, we found that miR-23b-5p could downregulate TAB1 levels, while co-transfection of hsa_circ_0021727 neutralized this effect. At the same time, the level of TAB1 was not affected by INH14 (Fig. 7N). This indicated that the expression of TAB1 was regulated by both hsa_circ_0021727 and miR-23b-5p. We also found that the expression levels of CyclinD1, MMP2, and MMP9 were positively regulated by hsa_circ_0021727 and negatively regulated by miR-23b-5p. However, the expression of P21 showed the opposite effect (Fig. 7N). Knockdown of *TAB1* reduced the protein levels of CyclinD1, MMP2, MMP9, and NF-κB (Fig. 7M). This suggested that these factors were not only regulated by hsa_circ_0021727, but also by TAB1. In general, hsa_circ_0021727 can activate TAB1/NFκB signaling via miR-23b-5p.

### Hsa_circ_0021727 promotes ESCC cell proliferation, invasion, and migration by activating the TAB1/NFκB pathway

**Fig. 8:**
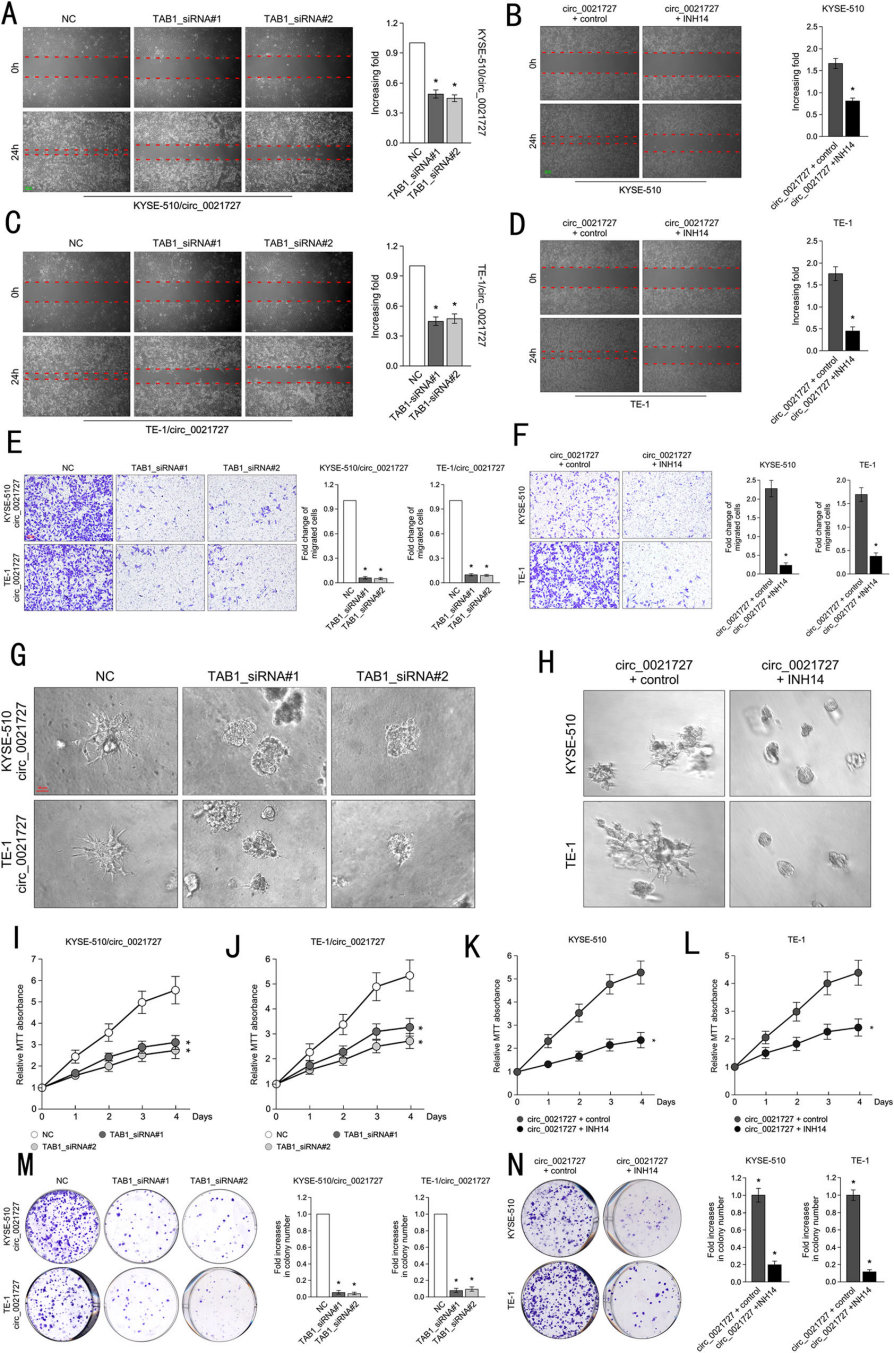
Hsa_circ_0021727 promotes ESCC cell proliferation, invasion, and migration by activating the TAB1/NFκB pathway. **A** and **C** Wound healing assays demonstrating the migration ability of KYSE510 and TE-1 cells overexpressing hsa_circ_0021727 or knocked down for *TAB1*. **B** and **D** Wound healing assays demonstrating the migration ability of KYSE510 and TE-1 cells overexpressing hsa_circ_0021727 and treated with INH14. **E** Transwell assays showing the invasion ability of KYSE510 cells and TE-1 cells overexpressing hsa_circ_0021727 or knocked down for *TAB1*. **F** Transwell assays showing the invasion ability of KYSE510 cells and TE-1 cells overexpressing hsa_circ_0021727 and treated with INH14. **G** Three-dimensional (3D) spheroid invasion assays revealed the invasion ability of KYSE510 and TE-1 cells overexpressing hsa_circ_0021727 or knocked down for *TAB1*. **H** 3D spheroid invasion assays demonstrating the invasion ability of KYSE510 and TE-1 cells overexpressing hsa_circ_0021727 and treated with INH14. **I** and **J** MTT assays showing the proliferation ability of KYSE510 and TE-1 cells overexpressing hsa_circ_0021727 or knocked down for *TAB1*. **K** and **L** MTT assays showing the proliferation ability of KYSE510 and TE-1 cells overexpressing hsa_circ_0021727 and treated with INH14. **M** Colony formation assays indicating the cell proliferation capability of KYSE510 and TE-1 cells overexpressing hsa_circ_0021727 or knocked down for *TAB1*. **N** Colony formation assays indicating the cell proliferation capability of KYSE510 and TE-1 cells overexpressing hsa_circ_0021727 and treated with INH14. Values are expressed as the means ± SD; *P < 0.05.

To investigate the effect of TAB1 on the function of ESCC cells, the *TAB1* knockdown plasmid and hsa_circ_0021727 overexpression vector were transfected into ESCC cells. Wound healing experiments showed that knockdown of *TAB1* increased the width of the linear wounds (Fig. 8A, C). After the addition of INH14, the migration ability of ESCC cells was inhibited (Fig. 8B, D). The results of Transwell assays showed that the invasive ability of ESCC cells was weakened after transfection with the *TAB1* knockdown plasmid (Fig. 8E). The addition of INH14 on the basis of overexpression of hsa_circ_0021727 also attenuated the invasion ability of ESCC cells (Fig. 8F). We obtained the same trend with the 3D spheroid invasion assays (Fig. 8G, H). The results of MTT experiments after co-transfection of the hsa_circ_0021727 overexpression vector and *TAB1* knockdown plasmid indicated that TAB1 attenuated the ability of hsa_circ_0021727 (Fig. 8I, J). INH14 also inhibited the ability of hsa_circ_0021727 (Fig. 8K, L). We verified these results using colony formation assays (Fig. 8M, N). In short, hsa_circ_0021727 promotes ESCC cell proliferation, invasion, and migration by activating the TAB1/NFκB pathway.

## Discussion

Esophageal cancer is characterized by high morbidity and mortality, ranking sixth in incidence and fifth in mortality [35]. Factors such as a hot diet and long-term nitrite intake increase the incidence of ESCC [36, 37]. ESCC is difficult to detect in the early stage, and the treatment effect is extremely poor. The deadly nature of ESCC means that the search for new biomarkers and targets has become an urgent issue. In this study, we discovered a novel circRNA that can serve as a potential biomarker and target for the early diagnosis and treatment of ESCC.

It has been widely reported that circRNAs play an important role in cancer. Their earliest discovered function is to negatively regulate miRNAs by acting as miRNA sponges. For example, hsa_circ_001783 promoted the progression of breast cancer via sponging miR-200c-3p [38]. CircRNA ciRS-7 accelerates ESCC progression by acting as an miR-876-5p sponge to enhance melanoma-associated antigen (MAGE-A) family expression [39]. CircRNAs were also found to be involved in the regulation of certain protein-protein and protein-RNA interactions [40, 41]. The general view that circRNA is a kind of noncoding RNA has been overturned. Some circRNAs are translated into polypeptides involved in tumor progression [42]. Although the functions of circRNAs have been gradually discovered, there are still many new functions worthy of exploration. In the present study, we found that hsa_circ_0021727 promoted ESCC progression. Its mechanism was closely related to the NFκB pathway. The NFκB pathway plays an important role in the occurrence and development of tumors [43, 44]. NF-κB signaling proteins were found to be upregulated in tissues from patients with ESCC [45]. Downregulation of p65 can increase tumor apoptosis and potentiates the effects of 5-fluorouracil by suppressing the NF-κB signaling pathway [46].Two NF-κB inhibitors (Bay11-7082 and sulfasalazine) were found to be potentially helpful in the treatment of ESCC [47]. CircRNAs have been found to modulate NF-κB signaling pathway under different settings [48]. CircRNA circIKBKB promotes breast cancer bone metastasis through sustaining NF-κB/bone remodeling factor signaling [49] CircRNA GLIS2 promotes colorectal cancer cell motility via activation of the NF-κB pathway [50]. Although the relationship between circRNAs and the NF-κB pathway has been reported many times, their mechanisms in ESCC have not been explored. In this study, we explored the mechanism by which hsa_circ_0021727 promotes ESCC progression by regulating the NF-κB pathway. Our results might provide new ideas for the treatment of ESCC.

MicroRNAs play an important role in the occurrence and development of tumors. For instance, microRNA-106a regulates autophagy-related cell death and epithelial-mesenchyme transition (EMT) by targeting tumor protein P53 inducible nuclear protein 1 (TP53INP1) in lung cancer with bone metastasis [51]. Tumor-derived exosomal miR-934 induces macrophage M2 polarization to promote liver metastasis of colorectal cancer [52]. Studies have shown that circRNAs might have a base sequence that binds to miRNAs, thereby act as a ceRNA to competitively bind to miRNAs, relieving the inhibitory effect of the miRNAs on their target genes, which then affects the occurrence and development of tumors [53]. Circ-ZEB1 promotes phosphatidylinositol-4,5-bisphosphate 3-kinase catalytic subunit alpha (PIK3CA) expression by sponging miR-199a-3p, which affects the proliferation and apoptosis of hepatocellular carcinoma [54]. Our previous experiments found that hsa_circ_0021727 could act as a sponge for miR-23b-5p. miR-23b is derived from the chromosomal region 9q22,32 encoding mi-23b/27b/24-1 [23]. It has been reported that miR-23b is highly correlated with TAB2, TAB3, and the NF-κB pathway [55]. In addition, miR-23b was identified to act both as an oncogene and as a tumor suppressor. Increased expression of miR-23b promoted lung cancer cells viability, migration, invasion, and EMT [56]. miR-23b inhibits cell migration and invasion through targeting phosphodiesterase 7A (PDE7A) in colon cancer cells [57]. miR-23b-5p, a variant of miR-23b, was shown to be an important regulatory molecule in tumorigenesis and development. Dysregulation of miR-23b-5p promotes cell proliferation via targeting forkhead box M1 (FOXM1) in hepatocellular carcinoma [58]. miR-23b-5p promotes chemosensitivity toward temozolomide by negatively regulating Toll-like receptor 4 (TLR4) in glioma [59]. In our experiments, miR-23b-5p played an important role as a tumor suppressor. It could reduce the translation of the downstream target gene *TAB1*, thereby inactivating the NFκB pathway. Although there have been some reports on the role of miR-23b-5p in tumors, its specific mechanism is still unclear. The downstream target genes affected by miR-23b-5p require further exploration.

TAB1 is an adapter protein that can bind to TAK1 to change its structure and activate it [60]. TAK1 is a member of the protein kinase complex consisting of TAK1, TAB1 and TAB2 that phosphorylates IKKβ and NF-kappa-beta-inducing Kinas (NIK). The TAK1-TABs complex phosphorylates IKKβ at Ser177 and Ser181, which is required for the activation of NF-κB signaling [61]. Therefore, TAB1 and TAK1 are key regulators of NF-κB activation. NF-κB is an important intracellular nuclear transcription factor complex, which has five members, including NF-kB1 (p50), NF-kB2 (p52), RelA (p65), RelB, and c-Rel [62]. Both the TAK1-TABs complex and NF-κB are considered to be highly tumor-related factors. TAB1 was reported to promoted cell proliferation, invasion, and migration in non-small cell lung cancer [63]. TAB1 also regulates papillary thyroid cancer cell proliferation and migration [64]. Santoro et al. identified TAK1 as a central hub integrating the most relevant signals sustaining pancreatic cancer aggressiveness and chemoresistance [65]. Tan et al. found that specific deletion of *TAK1* in hepatocytes promoted liver fibrosis and hepatocellular carcinoma [66]. Aberrant activation of the TAK1-TABs complex activates NF-κB signaling to promote tumor progression. For example, activation of the TAB1/TAK1/IKKβ/NF-κB signaling axis by TGF-β is a key factor in breast cancer cell invasion [67]. In this paper, the results showed that TAB1 expression correlated positively with the expression of hsa_circ_0021727 and negatively with the expression of miR-23b-5p. Moreover, activation of the TAB1/TAK1/IKKβ/NF-κB signaling axis induced proliferation, invasion, and migration of ESCC cells.

## Conclusion

**Fig. 9:**
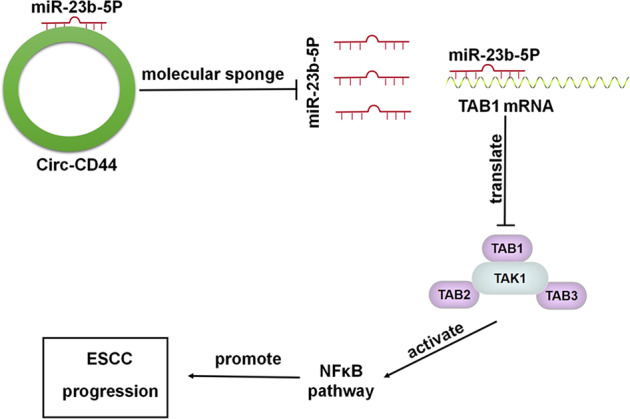
Circ-CD44 promotes ESCC progression by targeting miR-23b-5p to activate the TAB1/NFκB pathway.

In conclusion, we identified a novel circRNA (hsa_circ_0021727) that was aberrantly expressed in ESCC. The results indicated that hsa_circ_0021727 could promote the proliferation, invasion, and migration of ESCC cells. Critically, hsa_circ_0021727 sponged miR-23b-5p to activate the TAB1/NF-κB pathway (Fig. 9). The present study determined the mechanism of hsa_circ_0021727 in ESCC, providing biomarkers and potential targets for ESCC diagnosis and therapy.

## Supplementary information

Table S1

Table S2

Table S3

Table S4

supplementary legends

Figure S1

Figure S2

Figure S3

Figure S4

Figure S5

Figure S6

Figure S7

WB_Fig.7G

WB_Fig.7J

WB_Fig.7M

WB_Fig.7N

WB_Fig.S3C

WB_Fig.S4G

WB_Fig.S7

checklist

## Data Availability

The datasets used and/or analyzed during the current study are available from the corresponding author on reasonable request.
