# Correction: Hsa_circ_0021727 (circ-CD44) promotes ESCC progression by targeting miR-23b-5p to activate the TAB1/NFκB pathway

**DOI:** 10.1038/s41419-025-07437-y

**Published:** 2025-02-27

**Authors:** Fan Meng, Xiaokang Zhang, Yanting Wang, Jie Lin, Yulin Tang, Guisheng Zhang, Binqiang Qiu, Xingdu Zeng, Weiyou Liu, Xin He

**Affiliations:** 1 Digestive System Department, The First Affiliated Hospital of Gannan Medical University, Ganzhou, China; 2 Jiangxi Provincial Branch of China Clinical Medical Research Center for Geriatric Diseases, The First Affiliated Hospital of Gannan Medical University, Ganzhou, China; 3 Department of Respiratory and Critical Illness Medicine, The First Affiliated Hospital of Gannan Medical University, Ganzhou, China

**Keywords:** Cancer epigenetics, Tumour biomarkers

Correction to: *Cell Death and Disease* 10.1038/s41419-022-05541-x, published online 06 January 2023

It has come to our attention that there is an terrible error with exons location mode in Figure 1C. We have re-prepared the Figure 1C with right exons location of CD44 in corrected Figure 1. We sincerely request a corrigendum about the model and result description of Figure 1C in the page 3, result section: Please corrected “exon 6 to exon 9” as “exon 8 to exon 10”. We also unfortunately found a mistake about the relation between the representative image and the vertical axis in Figure 8N.

**Original Figure 1** 
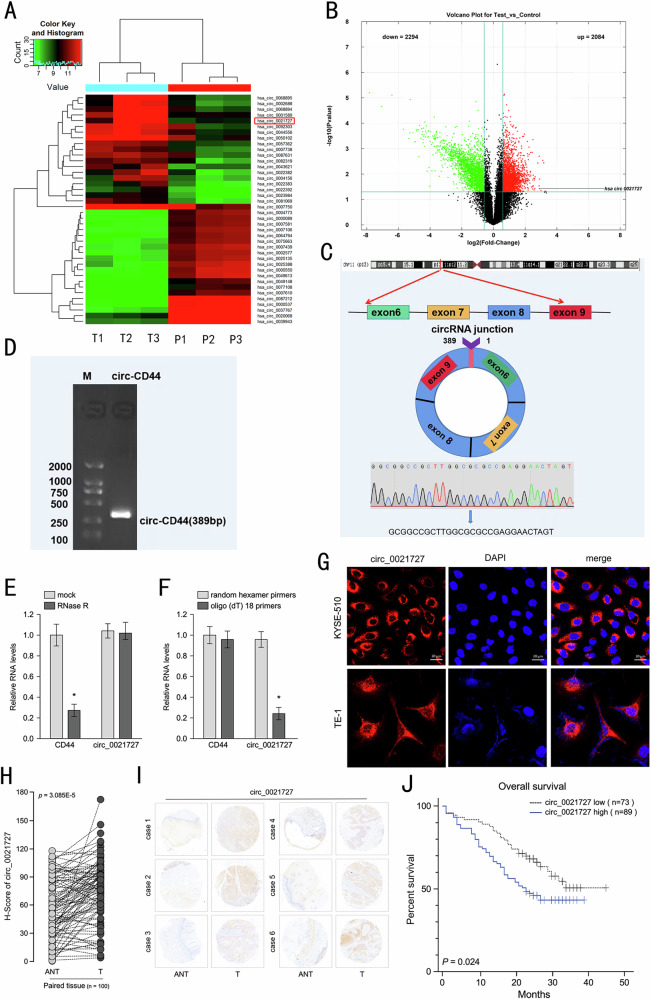


**Corrected Figure 1** 
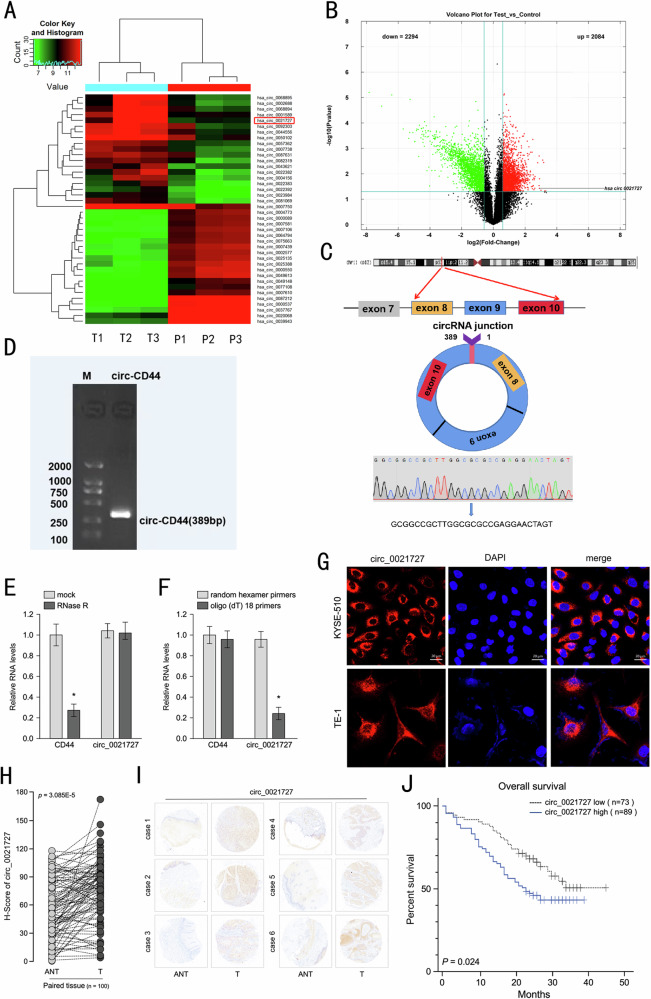


**Original Figure 8** 
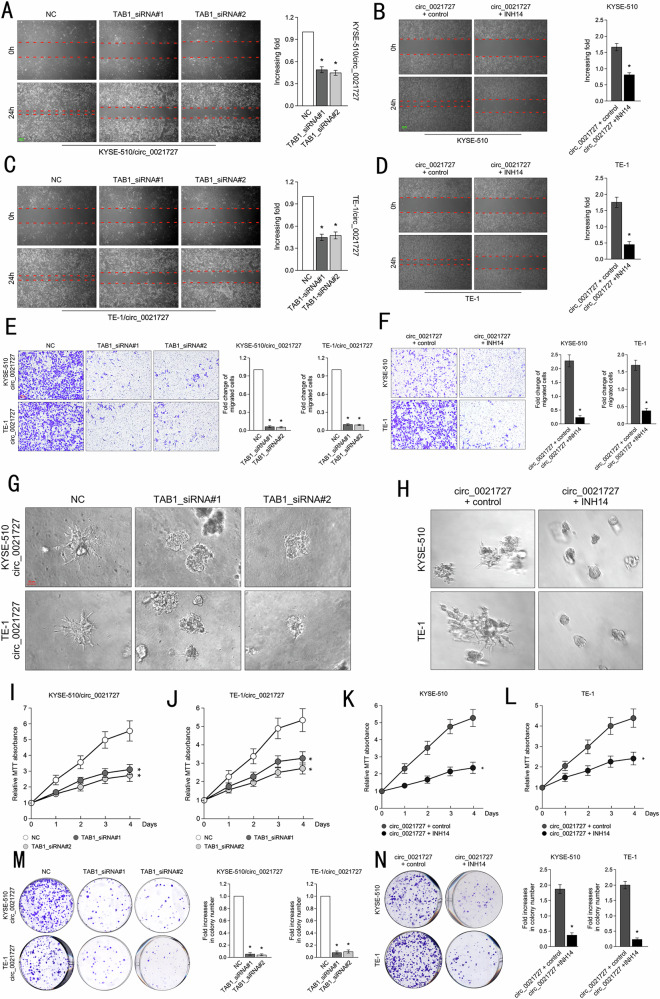


**Corrected Figure 8** 
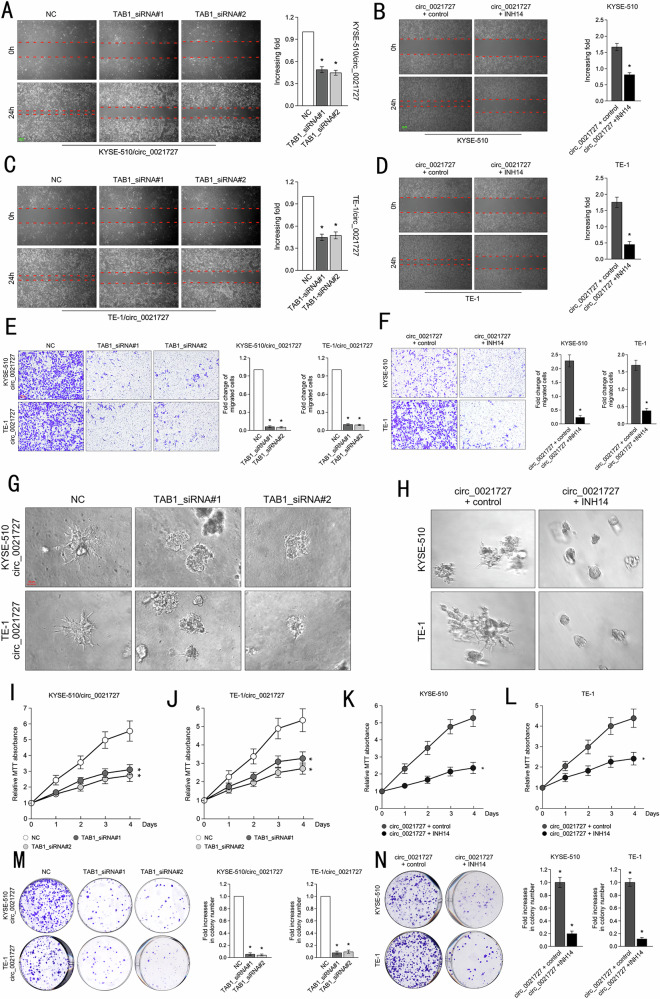


The original article has been corrected.

