# Supplementary material for: Hsa_circ_0021727 (circ-CD44) promotes ESCC progression by targeting miR-23b-5p to activate the TAB1/NFκB pathway
Source: Cell Death Dis. 2023 Jan 6;14(1):9. doi: 10.1038/s41419-022-05541-x (PMC9822936; doi:10.1038/s41419-022-05541-x)
Supplement: Supplementary file 1 — Table S1 [file 41419_2022_5541_MOESM1_ESM.doc]

**Table S1A. Clinicopathological characteristics of studied patients in esophageal cancer**

|  | **Number of cases** |
| --- | --- |
| **Age(years)** |  |
| > 55 | 132 |
| ≤ 55 | 30 |
| **AJCC clinical stage** |  |
| I | 3 |
| II | 76 |
| III | 81 |
| IV | 2 |
| **T classification** |  |
| T1 | 2 |
| T2 | 26 |
| T3 | 126 |
| T4 | 8 |
| **N classification** |  |
| N0 | 78 |
| N1 | 50 |
| N2 | 23 |
| N3 | 11 |
| **M classification** |  |
| Yes | 2 |
| No | 160 |
| **Gender** |  |
| Male | 140 |
| Female | 22 |
| **Survive or Mortality** |  |
| Survive | 83 |
| Mortality | 79 |

**Table S1B. The expression of circ_0021727 in ESCC**

| **Expression of circ_0021727** |  |
| --- | --- |
| Negative | 0 (0%) |
| Positive | 162 (100%) |
| Low expression | 73 (45.1%) |
| High expression | 89 (54.9%) |
