# Supplementary material for: Hsa_circ_0021727 (circ-CD44) promotes ESCC progression by targeting miR-23b-5p to activate the TAB1/NFκB pathway
Source: Cell Death Dis. 2023 Jan 6;14(1):9. doi: 10.1038/s41419-022-05541-x (PMC9822936; doi:10.1038/s41419-022-05541-x)
Supplement: Supplementary file 2 — Table S2 [file 41419_2022_5541_MOESM2_ESM.doc]

Table S2. Primers for RT-qPCR

| Primers | Sequences (5’-3’*) |  |
| --- | --- | --- |
| GAPDH:  CCND1: | F: GGAGCGAGATCCCTCCAAAAT  R:GGCTGTTGTCATACTTCTCATGG  F: GCTGCGAAGTGGAAACCATC  R: CCTCCTTCTGCACACATTTGAA |  |
| P21(CDKN1A)：  MMP2:  MMP9:  MYC(c-myc):  BCL2L1:  TWIST1:  VEGFA:  hsa-miR-23a-5p  hsa-miR-23b-5p  hsa-miR-218-5p  hsa-miR-433-3p  hsa-miR-494-5p  Hsa-circ-0021727  PGL3-basic-TAB1-3’UTR  PGL3-basic-TAB1-3’UTR-mut | F: TGTCCGTCAGAACCCATGC  R:AAAGTCGAAGTTCCATCGCTC  F: TACAGGATCATTGGCTACACACC  R: GGTCACATCGCTCCAGACT  F: TGTACCGCTATGGTTACACTCG  R:GGCAGGGACAGTTGCTTCT  F: GGCTCCTGGCAAAAGGTCA  R: CTGCGTAGTTGTGCTGATGT  F:GAGCTGGTGGTTGACTTTCTC  R:TCCATCTCCGATTCAGTCCCT  F:GTCCGCAGTCTTACGAGGAG  R:GCTTGAGGGTCTGAATCTTGCT  F:AGGGCAGAATCATCACGAAGT  R:AGGGTCTCGATTGGATGGCA  RT:ctcaactggtgtcgtggagtcggcaattcagttgagGGAAATCC  F:acactccagctgggATCACATTGCCAGGGATTT  RT:ctcaactggtgtcgtggagtcggcaattcagttgagGGAAATCC  F:acactccagctgggATCACATTGCCAGGGATTT  RT:ctcaactggtgtcgtggagtcggcaattcagttgagACATGGTT  F:acactccagctgggTTGTGCTTGATCTAACCA  RT:ctcaactggtgtcgtggagtcggcaattcagttgagACACCGAG  F:acactccagctgggATCATGATGGGCTCCTCG  RT:ctcaactggtgtcgtggagtcggcaattcagttgagGAGGTTTC  F:acactccagctgggTGAAACATACACGGGAAA  F:CACCACGGGCTTTTGACCAC  R:TGAATGAGGGGAGGGTGTGC  F:ctctcaagggcatcggtcgacAATGCAGCCCAAGCAGGG  R:gcgtgctagcccgggctcgagTCTAACTCAGGCCCTGGGCT  F:tgcatccagagtggaagaaaTCCTGCATCCAGAGTGGAACC  R:cttccactctggatgcaggaTGGGTTCCACTCTGGATGCA |  |
