# Supplementary material for: Hsa_circ_0021727 (circ-CD44) promotes ESCC progression by targeting miR-23b-5p to activate the TAB1/NFκB pathway
Source: Cell Death Dis. 2023 Jan 6;14(1):9. doi: 10.1038/s41419-022-05541-x (PMC9822936; doi:10.1038/s41419-022-05541-x)
Supplement: Supplementary file 3 — Table S3 [file 41419_2022_5541_MOESM3_ESM.doc]

| Targets | Sequences (5’-3’*) |  |
| --- | --- | --- |
| hsa_circ_0021727-1:  hsa_circ_0021727-2: | F:GATCCGGCACCACTGCTTATGAAGGACTCGAGTCCTTCATAAGCAGTGGTGCCTTTTTG  F:GATCCGCAACTCCTAGTAGTACAACGCTCGAGCGTTGTACTACTAGGAGTTGCTTTTTG |  |
| hsa_circ_0021727-2: | F:GATCCGCATGAGGGATATCGCCAAACCTCGAGGTTTGGCGATATCCCTCATGCTTTTTG |  |

Table S3A. Primer sequences of shRNAs for construction of stably-transfected cell

Table S3B.Various RNA probe sequences

| Targets | Sequences (5’-3’*) |  |
| --- | --- | --- |
| hsa-circ-0021727(ISH probe ) : | TGGTGTGGTTGAAACAGCTGTCCCTGTTGTCG |  |
| hsa_circ_0021727( Fish probe )：  hsa-miR-23b-5p( Fish probe )：  Biotin(RNA pulldown probe ):  hsa-miR-23a-5p(RNA pulldown probe ):  hsa-miR-23b-5p(RNA pulldown probe ):  hsa-miR-218-5p(RNA pulldown probe ):  hsa-miR-433-3p(RNA pulldown probe ):  hsa-miR-494-5p(RNA pulldown probe ): | GTGGTTGAAACAGCTGTCCCTGTTGTCGAATG  UGGGUUCCUGGCAUGCUGAUUU  GAAACAGCTGTCCCTGTTGTCGAATGGGAGTCTTC  GGGGUUCCUGGGGAUGGGAUUU  UGGGUUCCUGGCAUGCUGAUUU  UUGUGCUUGAUCUAACCAUGU  AUCAUGAUGGGCUCCUCGGUGU  AGGUUGUCCGUGUUGUCUUCUCU |  |
