# Supplementary material for: Hsa_circ_0021727 (circ-CD44) promotes ESCC progression by targeting miR-23b-5p to activate the TAB1/NFκB pathway
Source: Cell Death Dis. 2023 Jan 6;14(1):9. doi: 10.1038/s41419-022-05541-x (PMC9822936; doi:10.1038/s41419-022-05541-x)
Supplement: Supplementary file 4 — Table S4 [file 41419_2022_5541_MOESM4_ESM.doc]

Table S4. Antibody concentration for Western blot analysis

| Antibody | concentration |  |
| --- | --- | --- |
| TAB1(Abcam,Cambridge, MA):  Cyclin D1(Abcam,Cambridge, MA): | 1:500  1:1000 |  |
| p21(Abcam,Cambridge, MA)：  MMP2(Abcam,Cambridge, MA):  MMP9(Abcam,Cambridge, MA):  p-IKKα(Abcam,Cambridge, MA):  p-IKKβ(Abcam,Cambridge, MA):  IKKα(Abcam,Cambridge, MA):  IKKβ(Abcam,Cambridge, MA):  p-P65(Abcam,Cambridge, MA):  P65(Abcam,Cambridge, MA):  GAPDH(Abcam,Cambridge, MA): | 1:1000  1:1000  1:2000  1:1000  1:1000  1:1000  1:1000  1:1000  1:1000  1:2000 |  |
