# Supplementary material for: Hsa_circ_0021727 (circ-CD44) promotes ESCC progression by targeting miR-23b-5p to activate the TAB1/NFκB pathway
Source: Cell Death Dis. 2023 Jan 6;14(1):9. doi: 10.1038/s41419-022-05541-x (PMC9822936; doi:10.1038/s41419-022-05541-x)
Supplement: Supplementary file 5 — supplementary legends [file 41419_2022_5541_MOESM5_ESM.doc]

**Supplemental Figure Legends**

**Fig. S1** The mRNA expression of hsa_circ_0021727 in Het-1A, TE-1 and KYSE-510 cell lines.Values are expressed as the means ± SD; *P < 0.05.

**Fig. S2** Bioinformatics analysis of hsa_circ_0021727, miR-23b-5p and TAB1 in ESCC datasets.**A** The expression of hsa_circ_0021727 in GSE dataset.**B** The expression of miR-23b-5p in TGCA.**C** The relationship between miR-23b-5p expression and survival of patients with esophageal cancer.**D** The relationship between TAB1 expression and survival of patients with esophageal cancer.Values are expressed as the means ± SD; *P < 0.05.

**Fig. S3** The expression of hsa_circ_0021727, miR-23b-5p, TAB1, Ki67 and MMP9 in mouse tumor tissues.**A** qRT-PCR detection of the expression hsa_circ_0021727 in mouse tumor tissues.**B** qRT-PCR detection of the expression miR-23b-5p in mouse tumor tissues.Values are expressed as the means ± SD; *P < 0.05.

**Fig. S4** The transfection efficiency of hsa_circ_0021727 and miR-23b-5p in ESCC cells**/**mouse tumor tissues.**A** and **B** qRT-PCR detection of the expression miR-23b-5p in KYSE510 cells and TE-1 cells co-transfected with miR-23b-5p and hsa_circ_0021727 overexpression vectors.**C** and **D** qRT-PCR detection of the expression hsa_circ_0021727 in KYSE510 cells and TE-1 cells co-transfected with miR-23b-5p and hsa_circ_0021727 overexpression vectors.**E** and **F** qRT-PCR detection of the expression hsa_circ_0021727 and miR-23b-5p in mouse tumor tissues.**G** Western blotting analysis detection of the protein expression TAB1 in mouse tumor tissues.Values are expressed as the means ± SD; *P < 0.05.

**Fig. S5** mRNA-seq analysis of expression of NFκB signaling related genes in ESCC cells transfected with hsa_circ_0021727 overexpression vectors or empty vector.

**Fig. S6** mRNA-seq analysis of expression TAB1 in ESCC cells transfected with hsa_circ_0021727 overexpression vectors or empty vector.Values are expressed as the means ± SD; *P < 0.05.

**Fig. S7** Western blotting analysis detection of the protein expression TAB1 in the TE-1 cell line overexpressing hsa_circ_0021727 and treated with INH14.
