# Supplementary figures and images for: Hsa_circ_0021727 (circ-CD44) promotes ESCC progression by targeting miR-23b-5p to activate the TAB1/NFκB pathway
Source: Cell Death Dis. 2023 Jan 6;14(1):9. doi: 10.1038/s41419-022-05541-x (PMC9822936; doi:10.1038/s41419-022-05541-x)

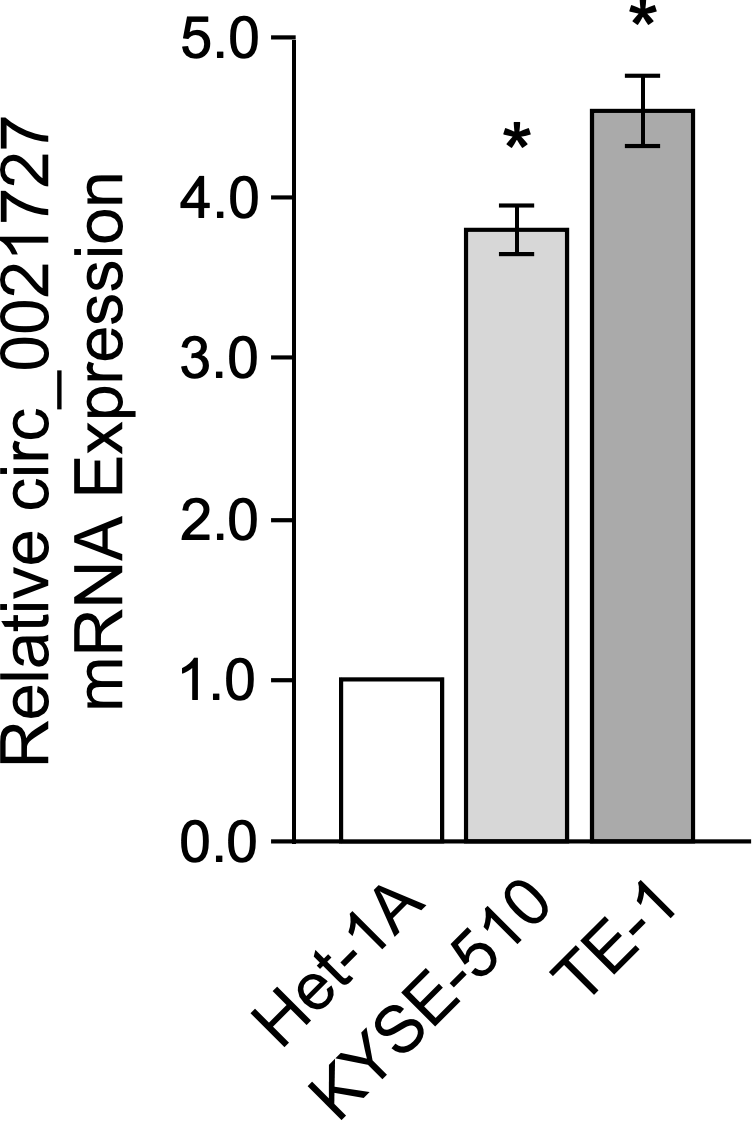

Supplement: Supplementary file 6 — Figure S1 [file 41419_2022_5541_MOESM6_ESM.tif]

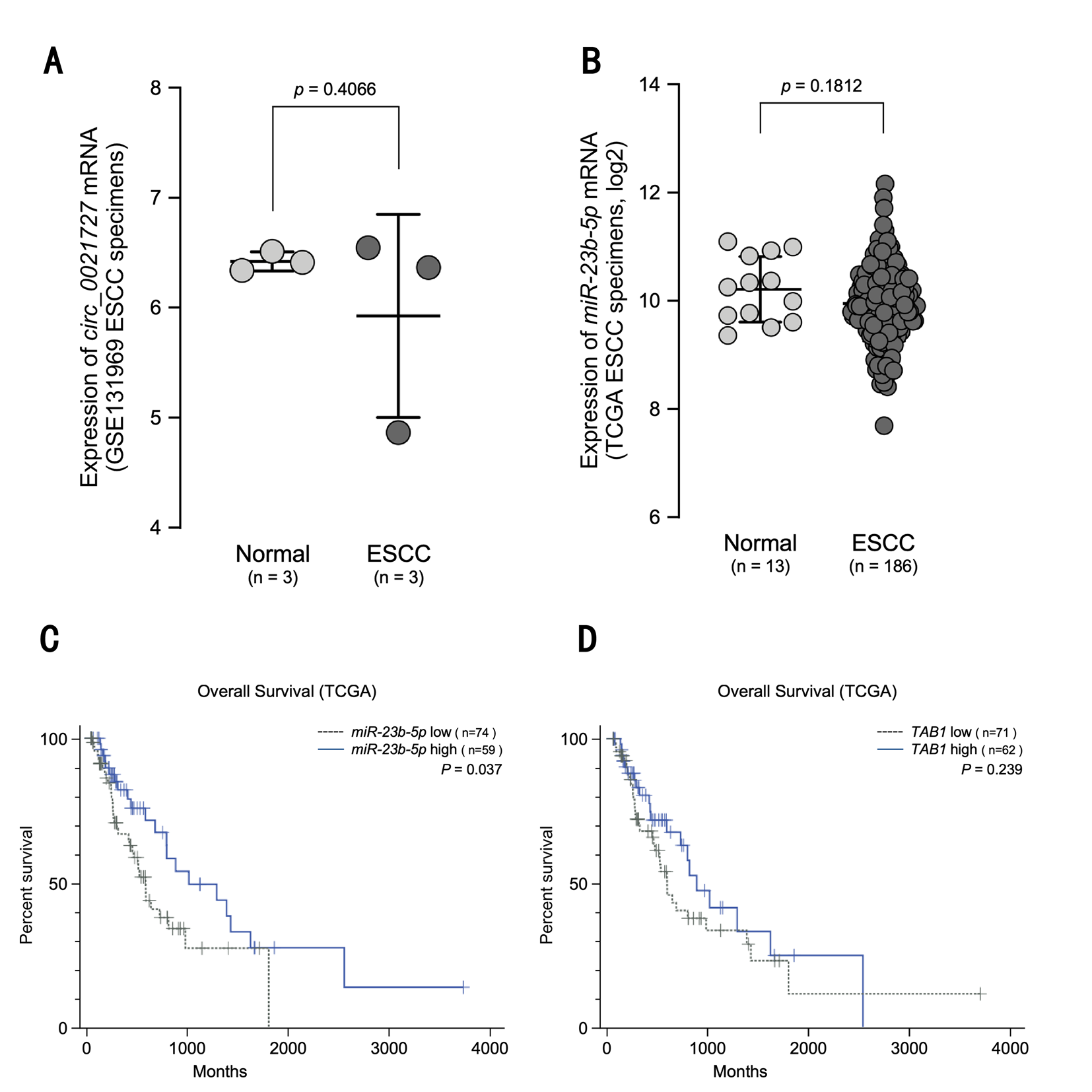

Supplement: Supplementary file 7 — Figure S2 [file 41419_2022_5541_MOESM7_ESM.tif]

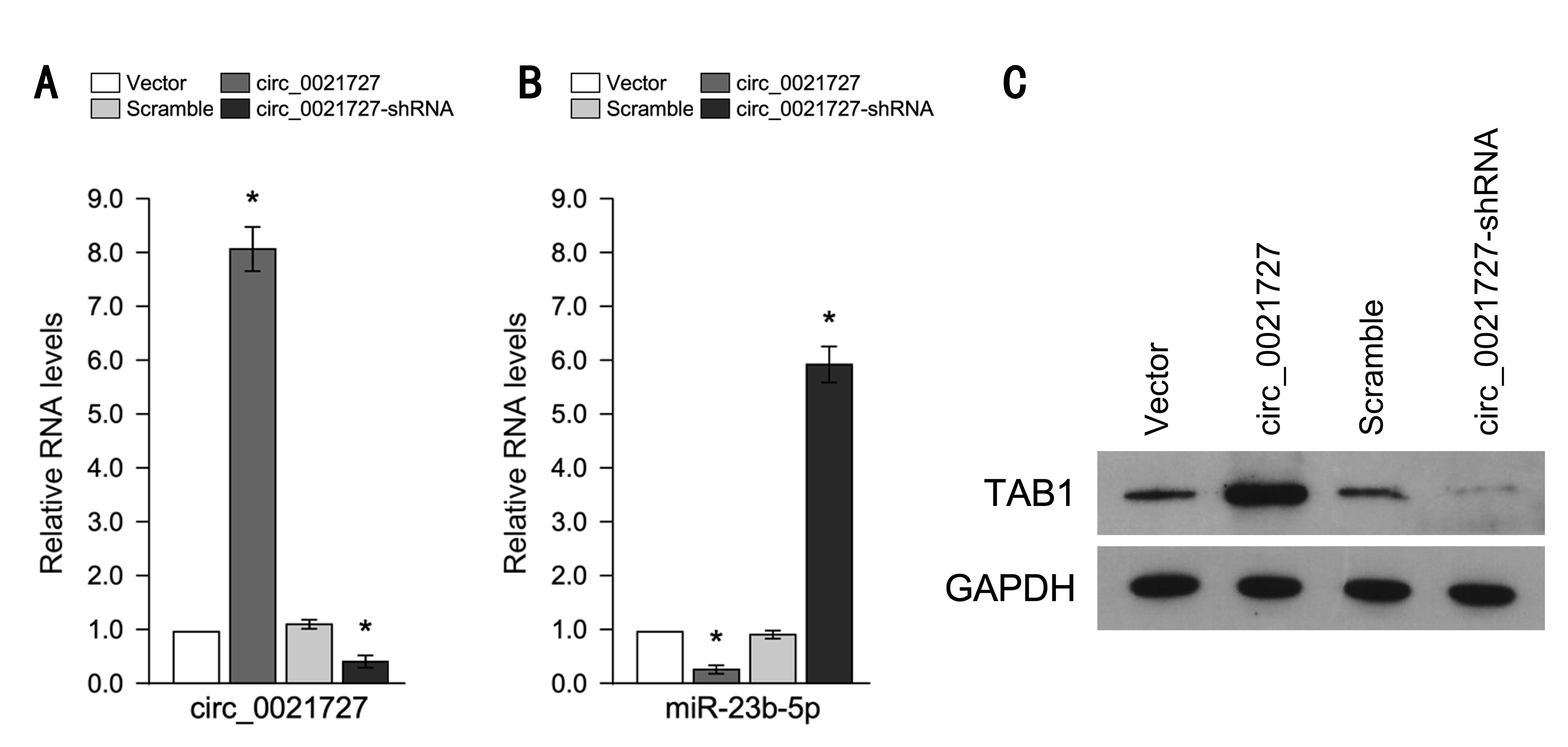

Supplement: Supplementary file 8 — Figure S3 [file 41419_2022_5541_MOESM8_ESM.tif]

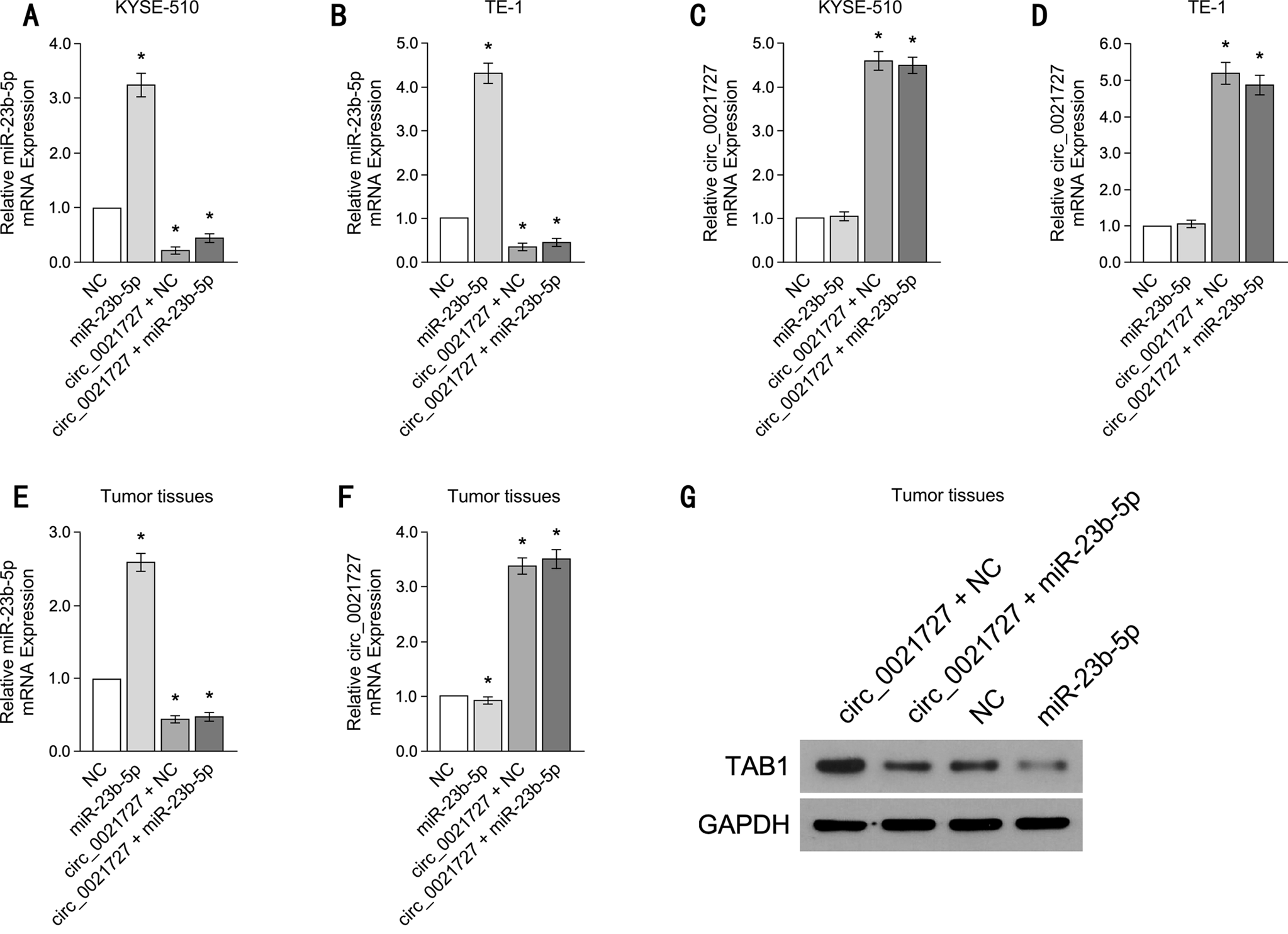

Supplement: Supplementary file 9 — Figure S4 [file 41419_2022_5541_MOESM9_ESM.tif]

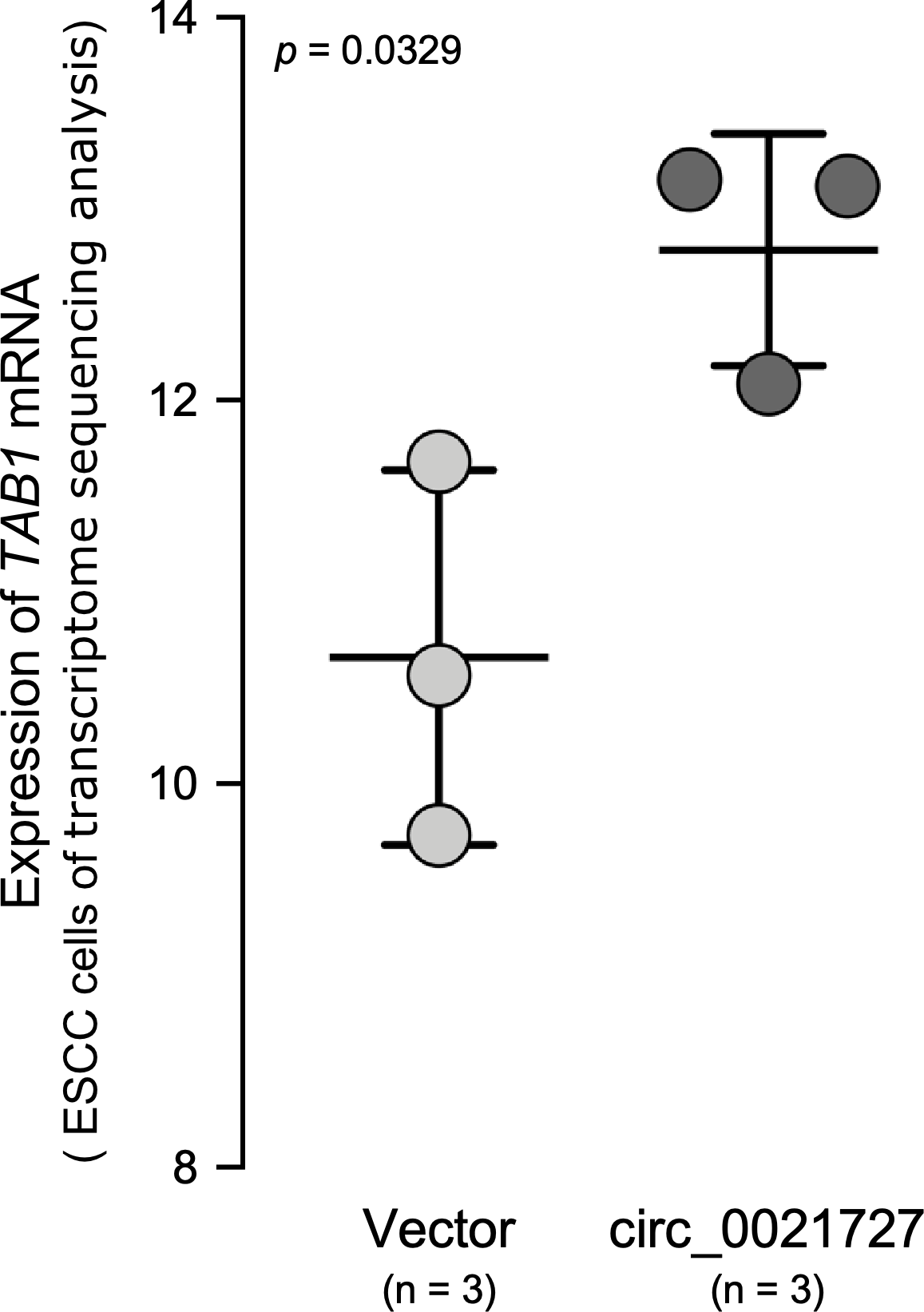

Supplement: Supplementary file 11 — Figure S6 [file 41419_2022_5541_MOESM11_ESM.tif]

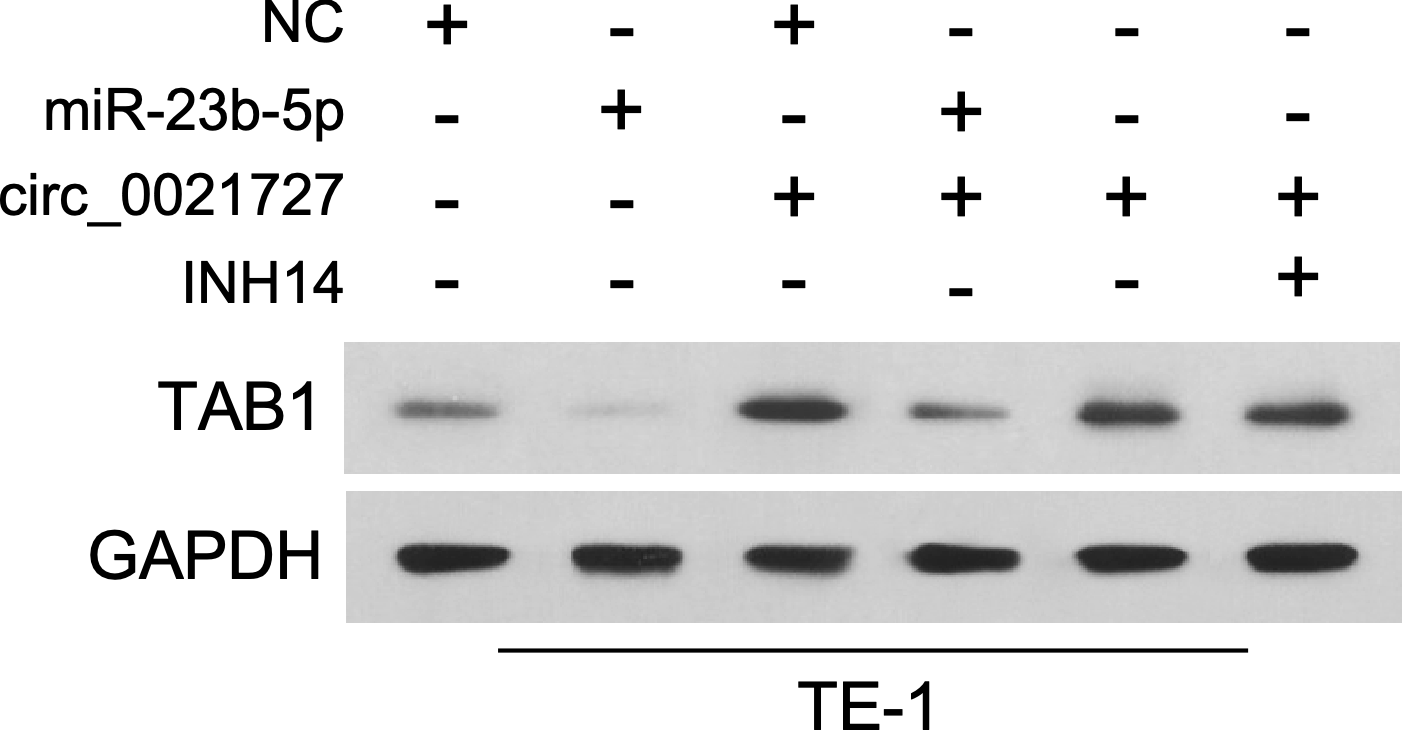

Supplement: Supplementary file 12 — Figure S7 [file 41419_2022_5541_MOESM12_ESM.tif]

...EΠΓ]•BXH•(2AFETΛ)•••

p-IKK-B

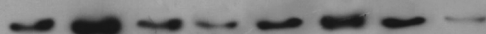

p-IkBa

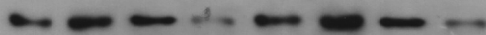

TAB1

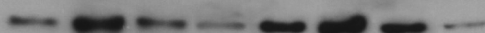

...FΠΓ]•BXH•(2AFETΛ)•••

GAPDH

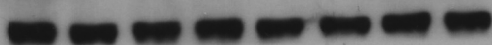

IKK-B

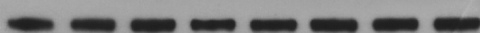

IκBa

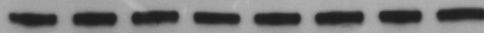

Supplement: Supplementary file 13 — WB_Fig.7G [file 41419_2022_5541_MOESM13_ESM.pdf]

TAB1

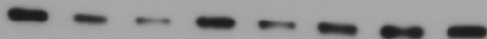

GAPDH

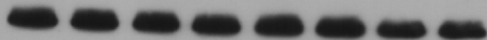

Supplement: Supplementary file 14 — WB_Fig.7J [file 41419_2022_5541_MOESM14_ESM.pdf]

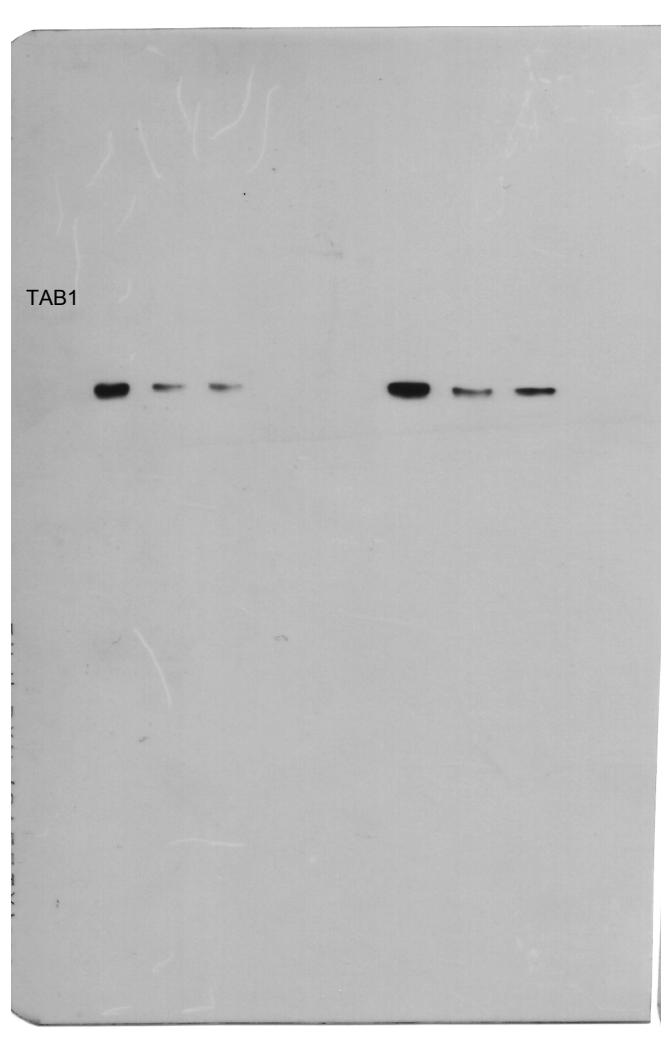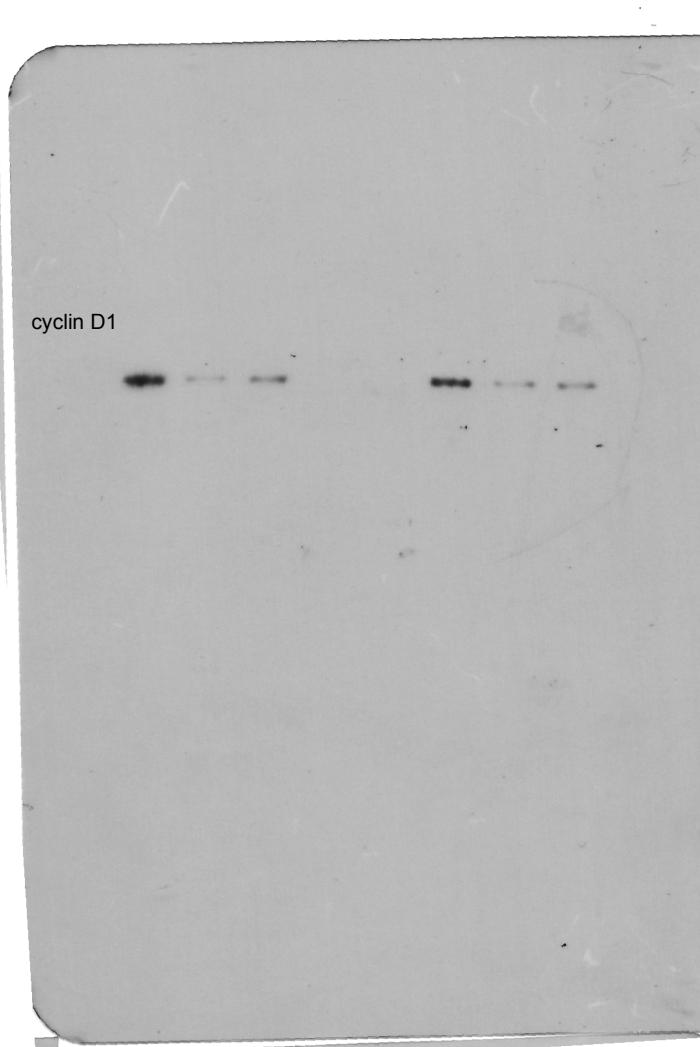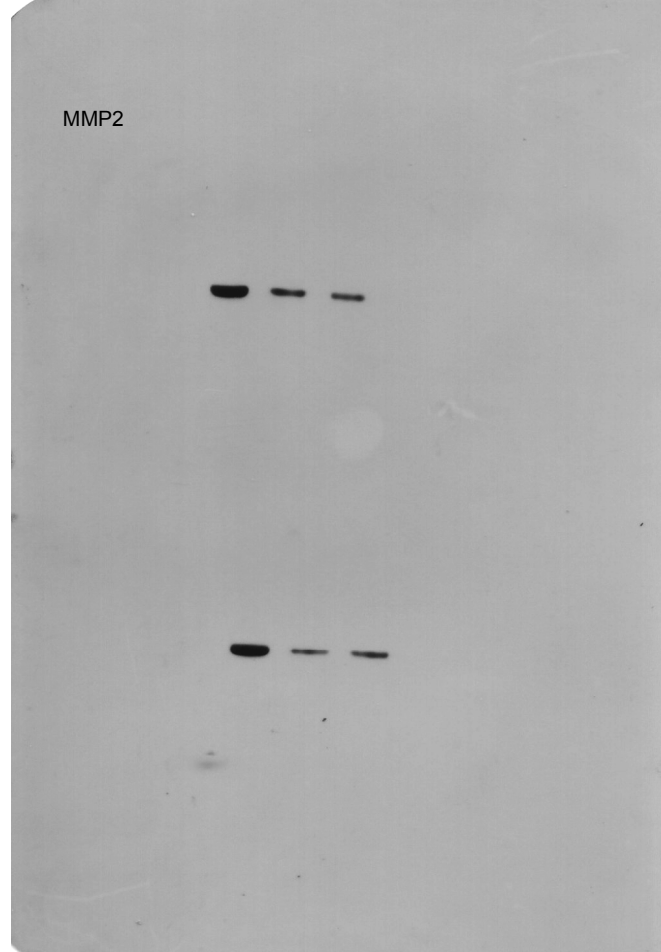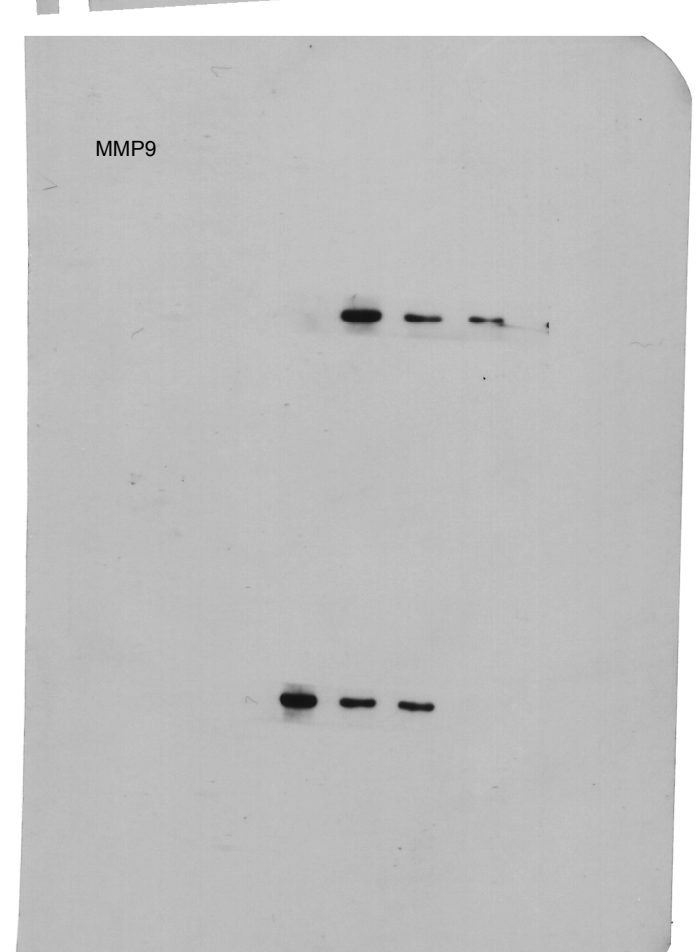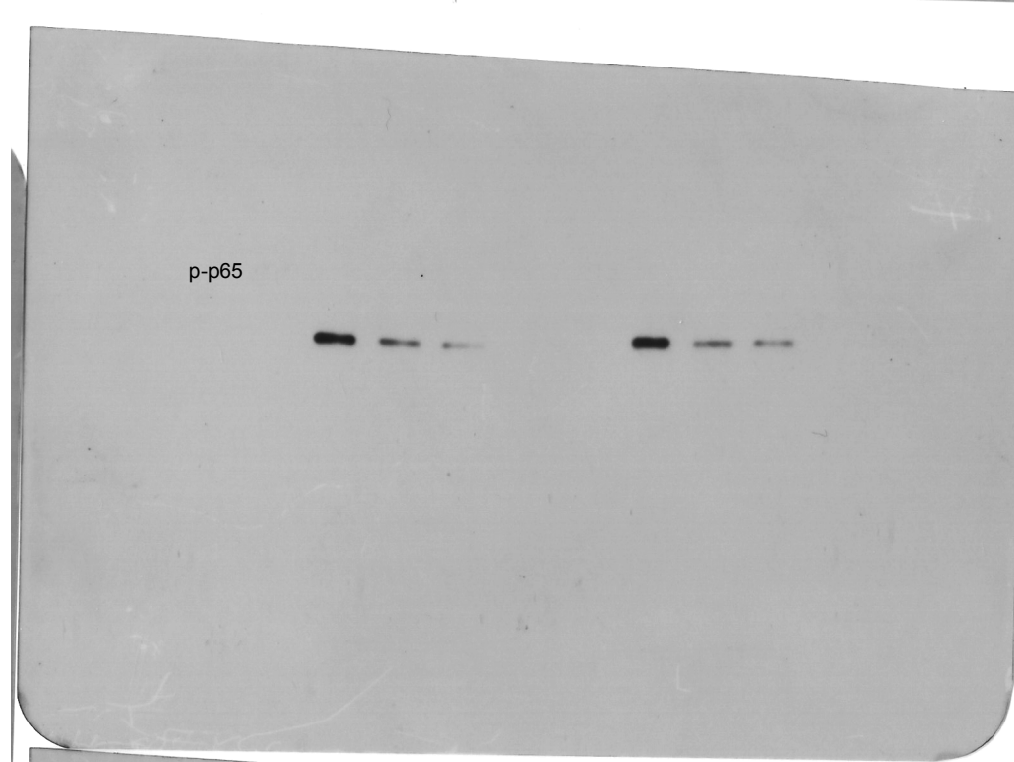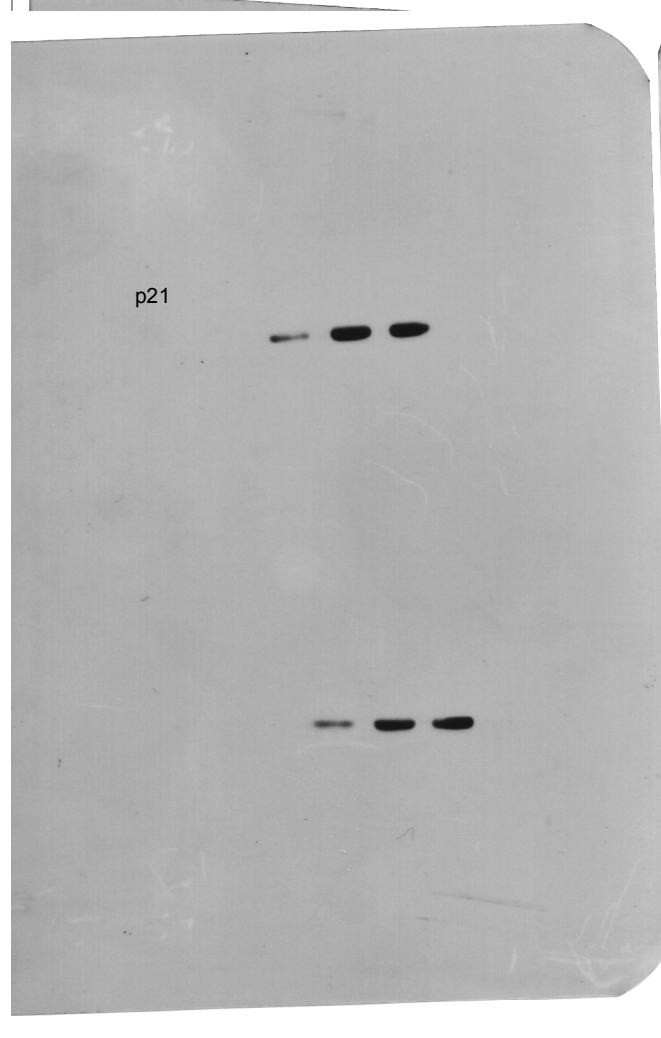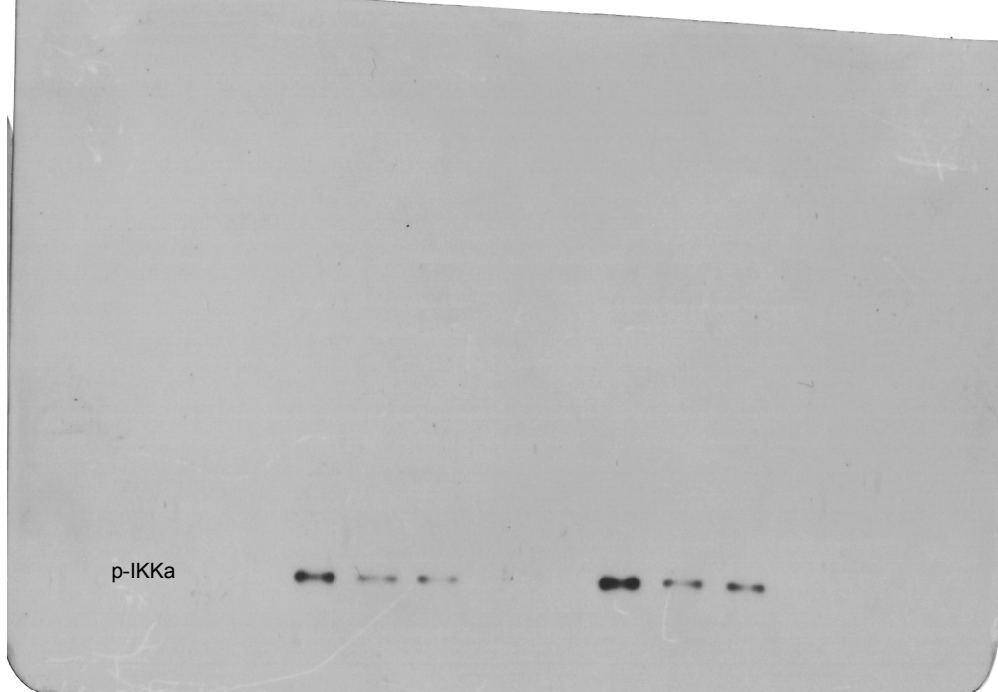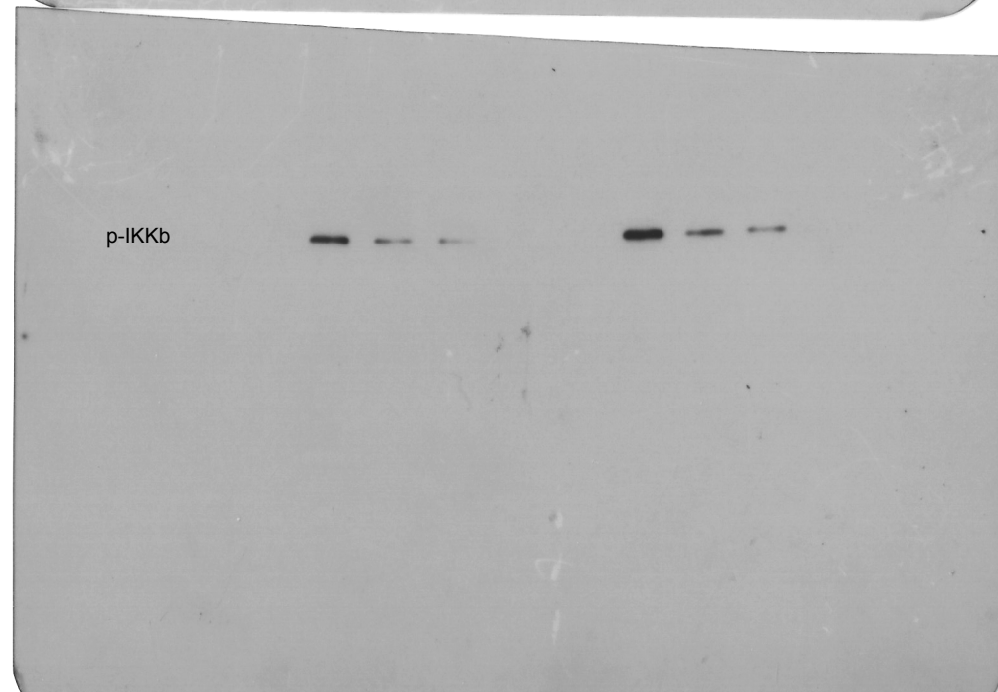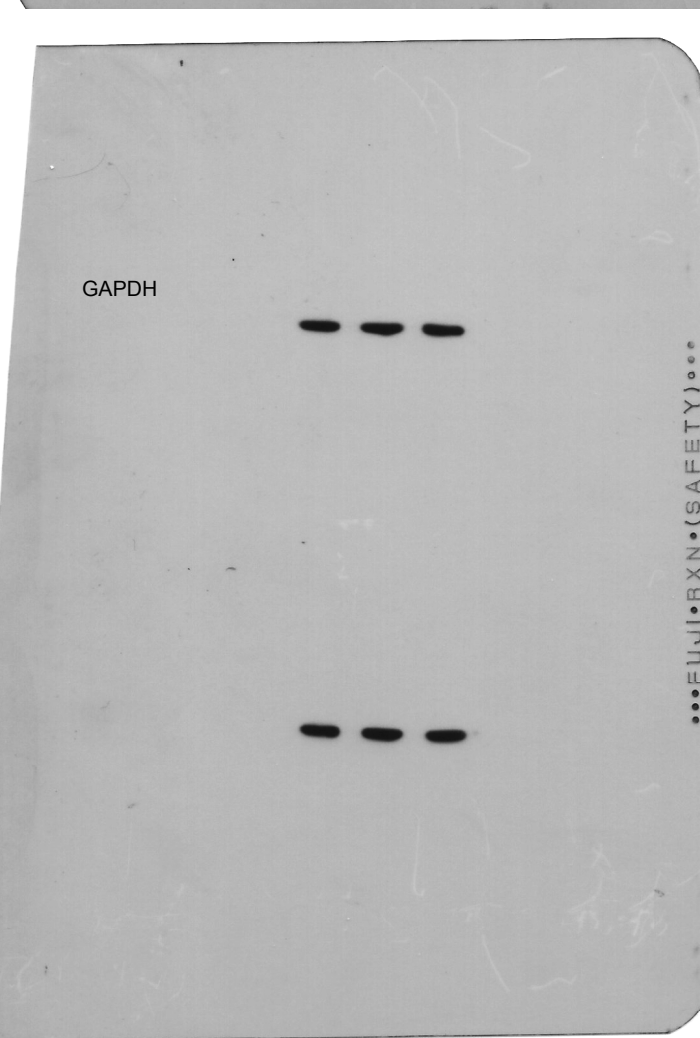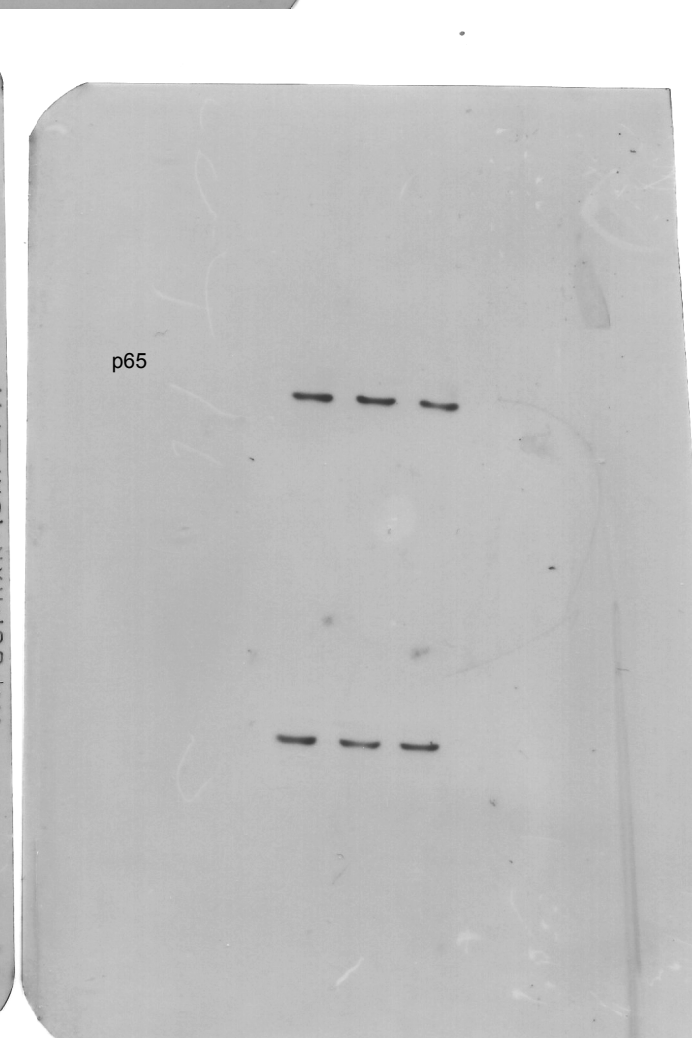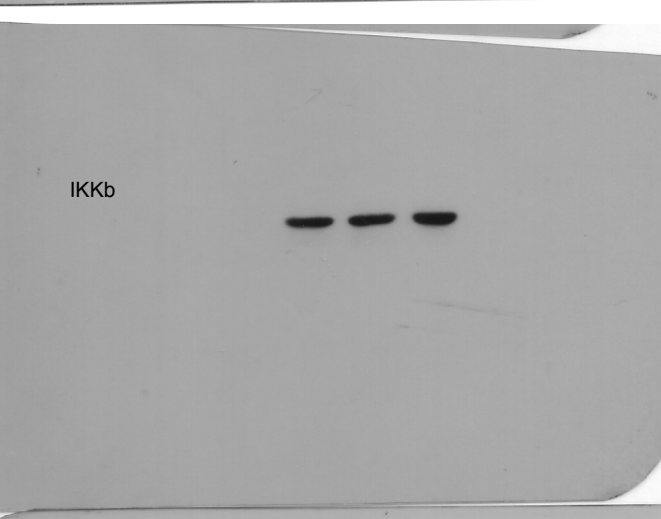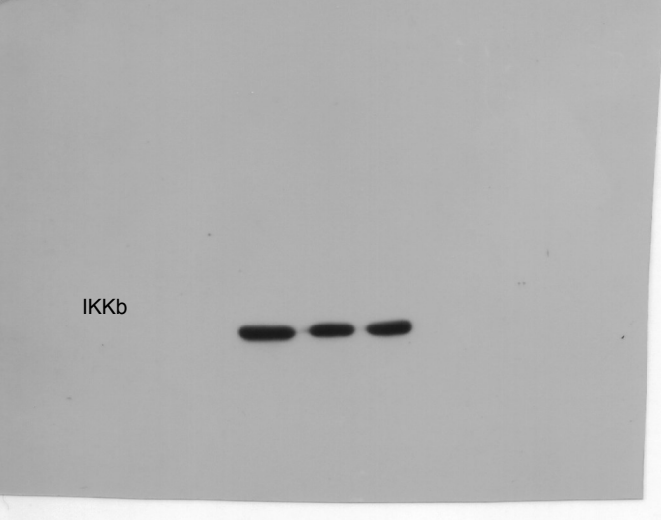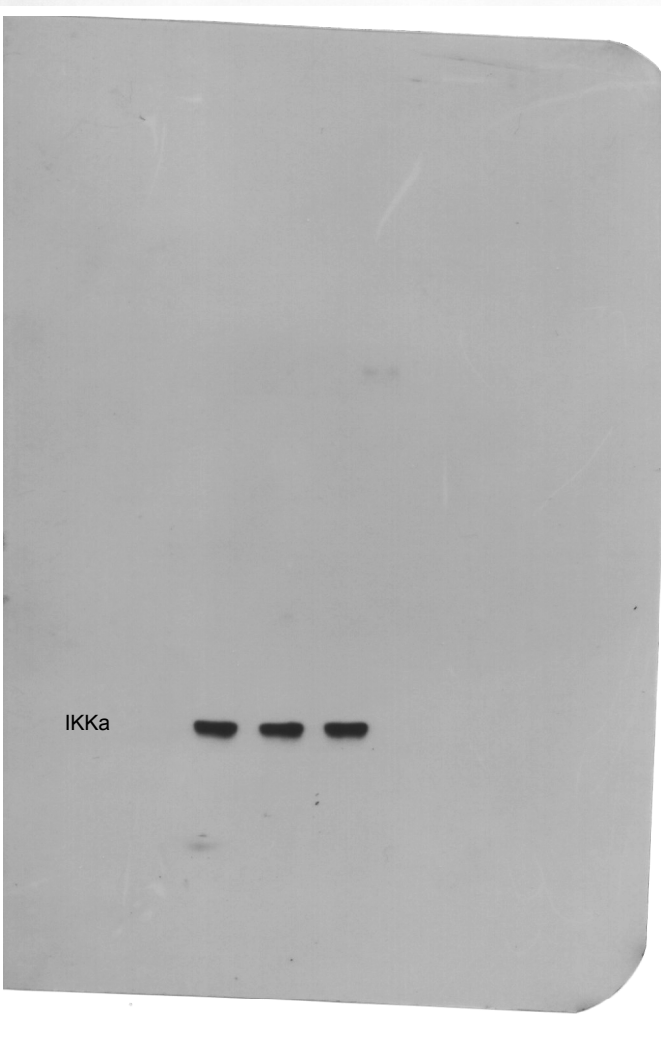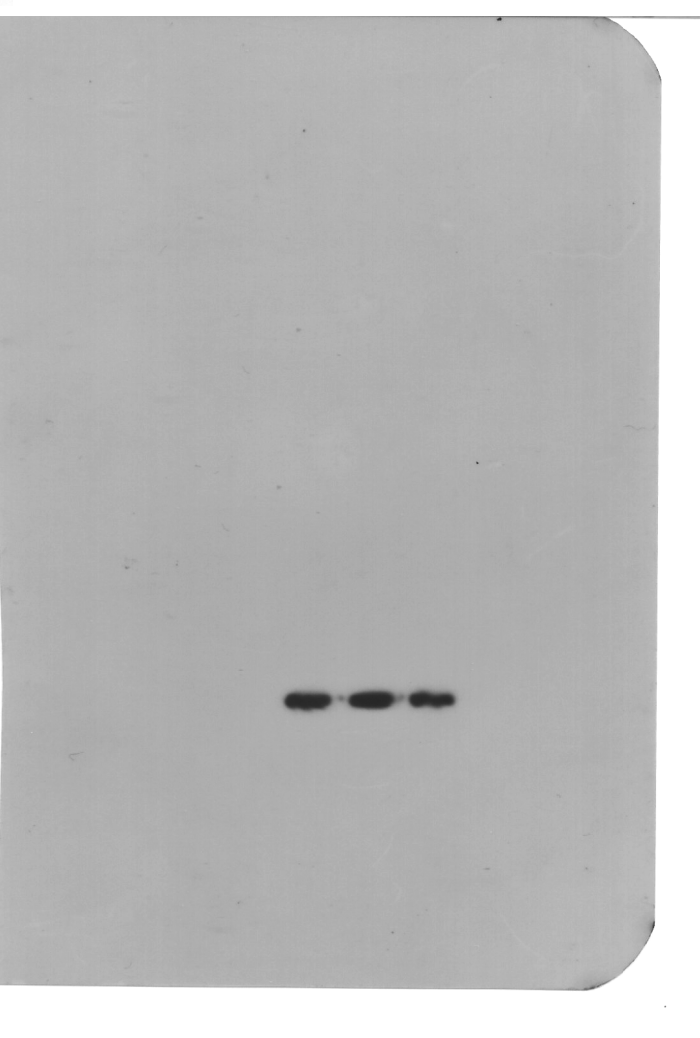

Supplement: Supplementary file 15 — WB_Fig.7M [file 41419_2022_5541_MOESM15_ESM.pdf]

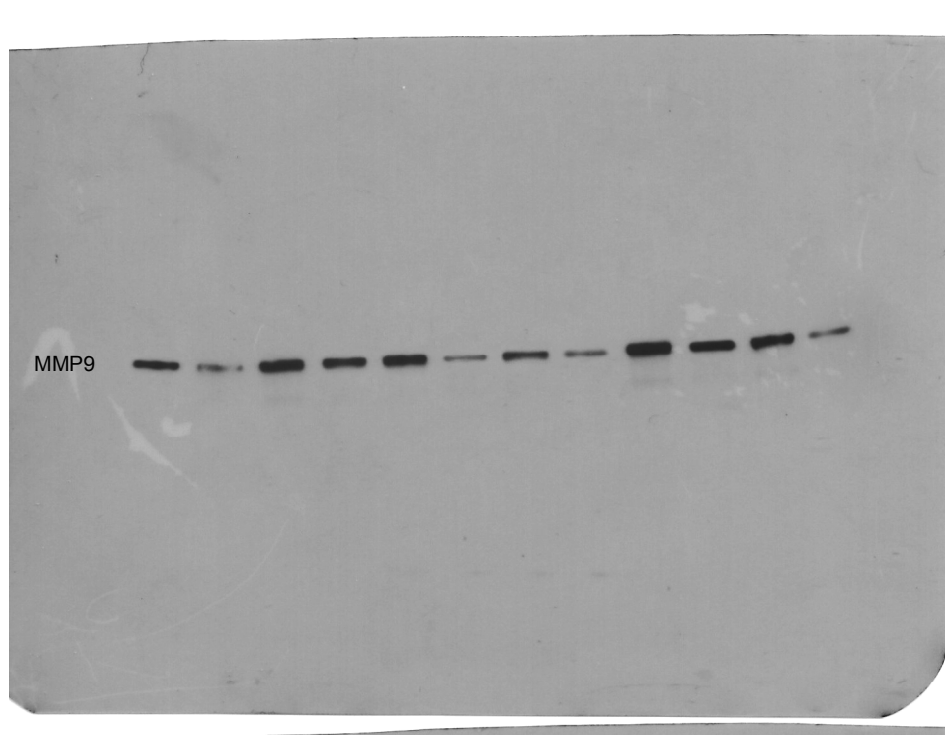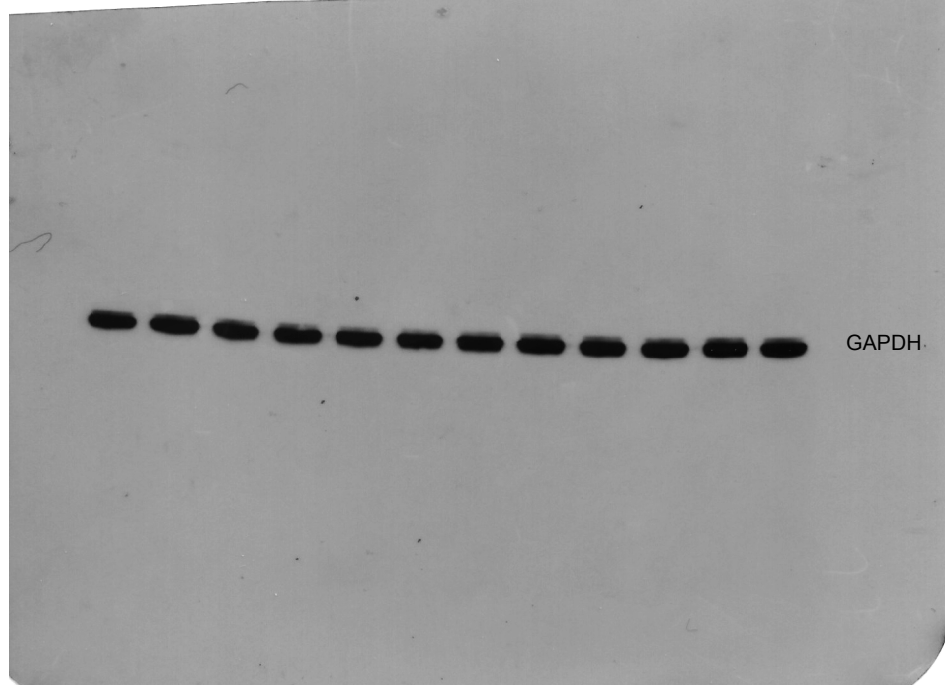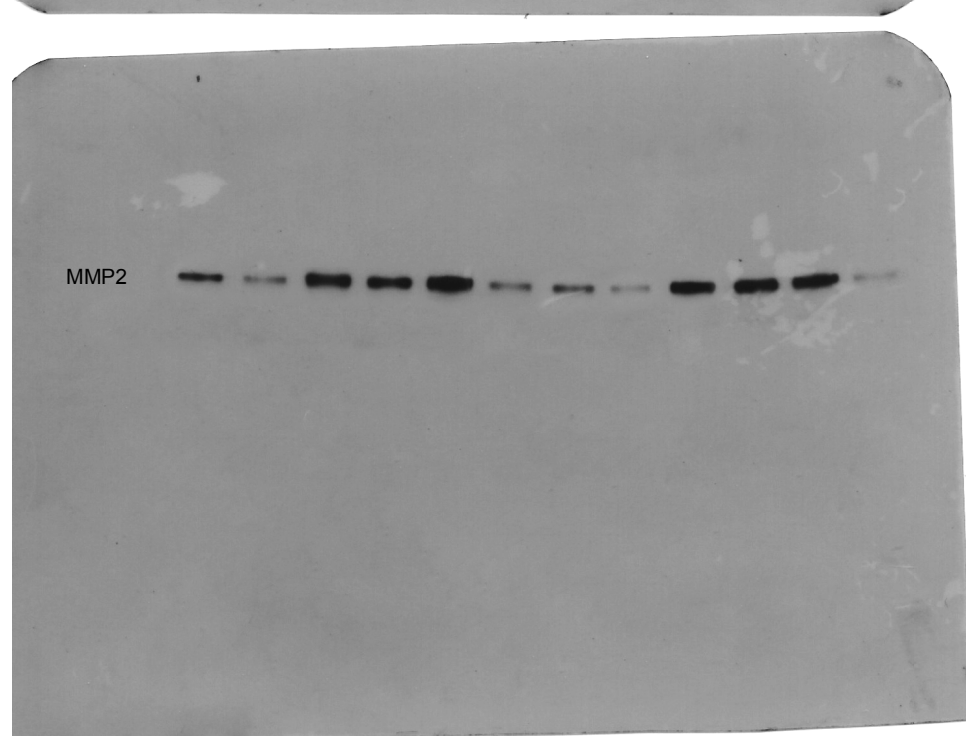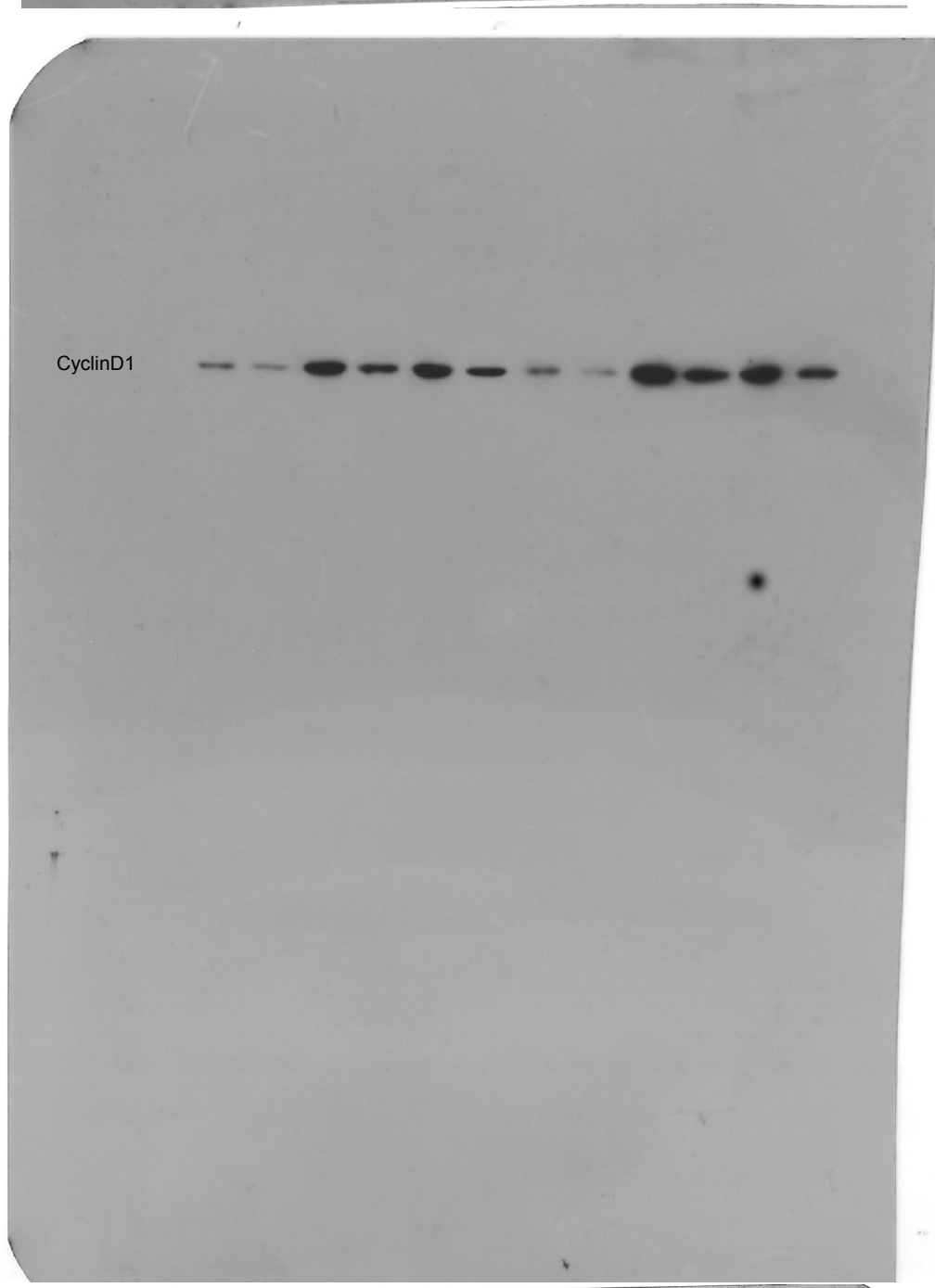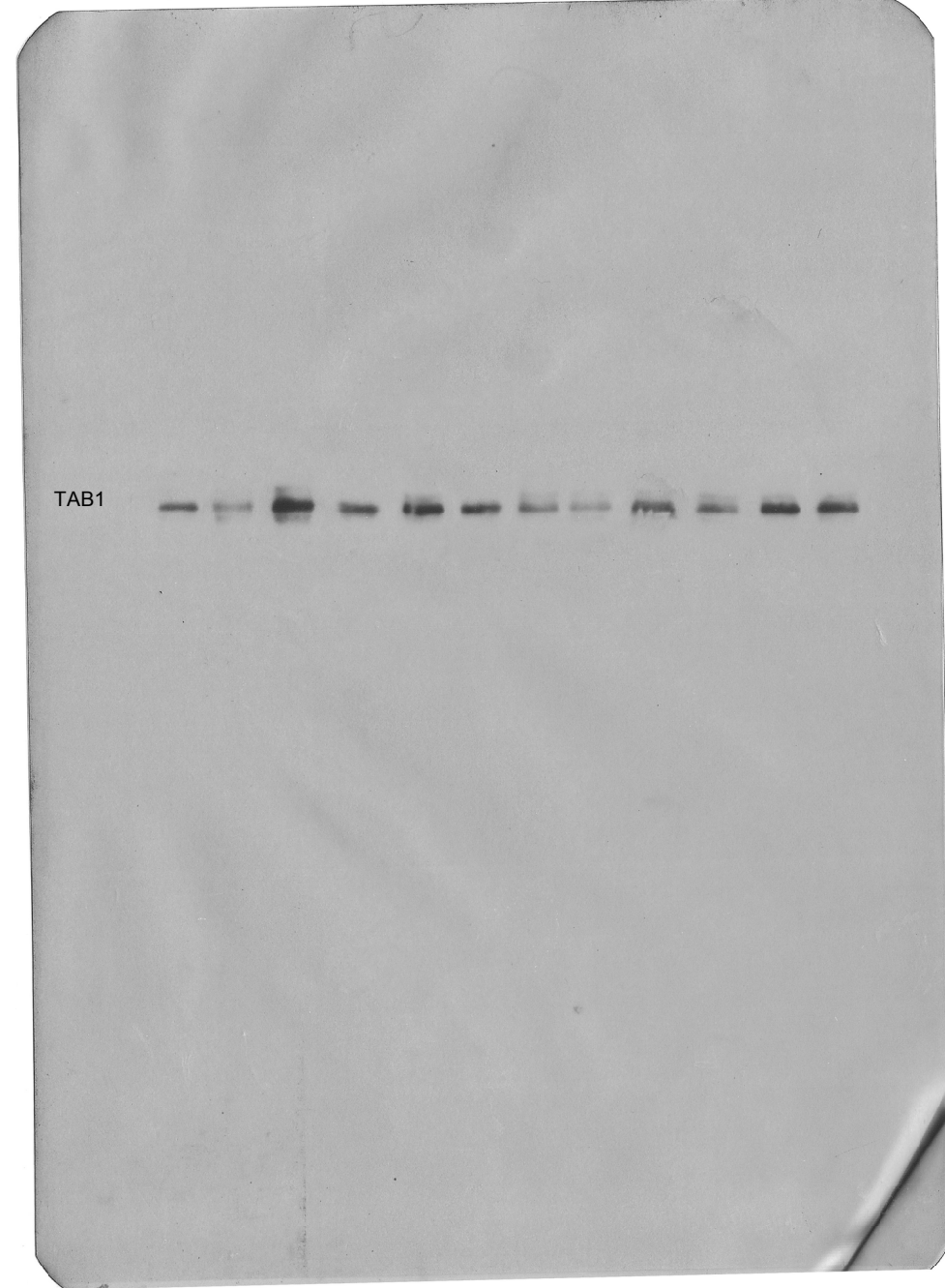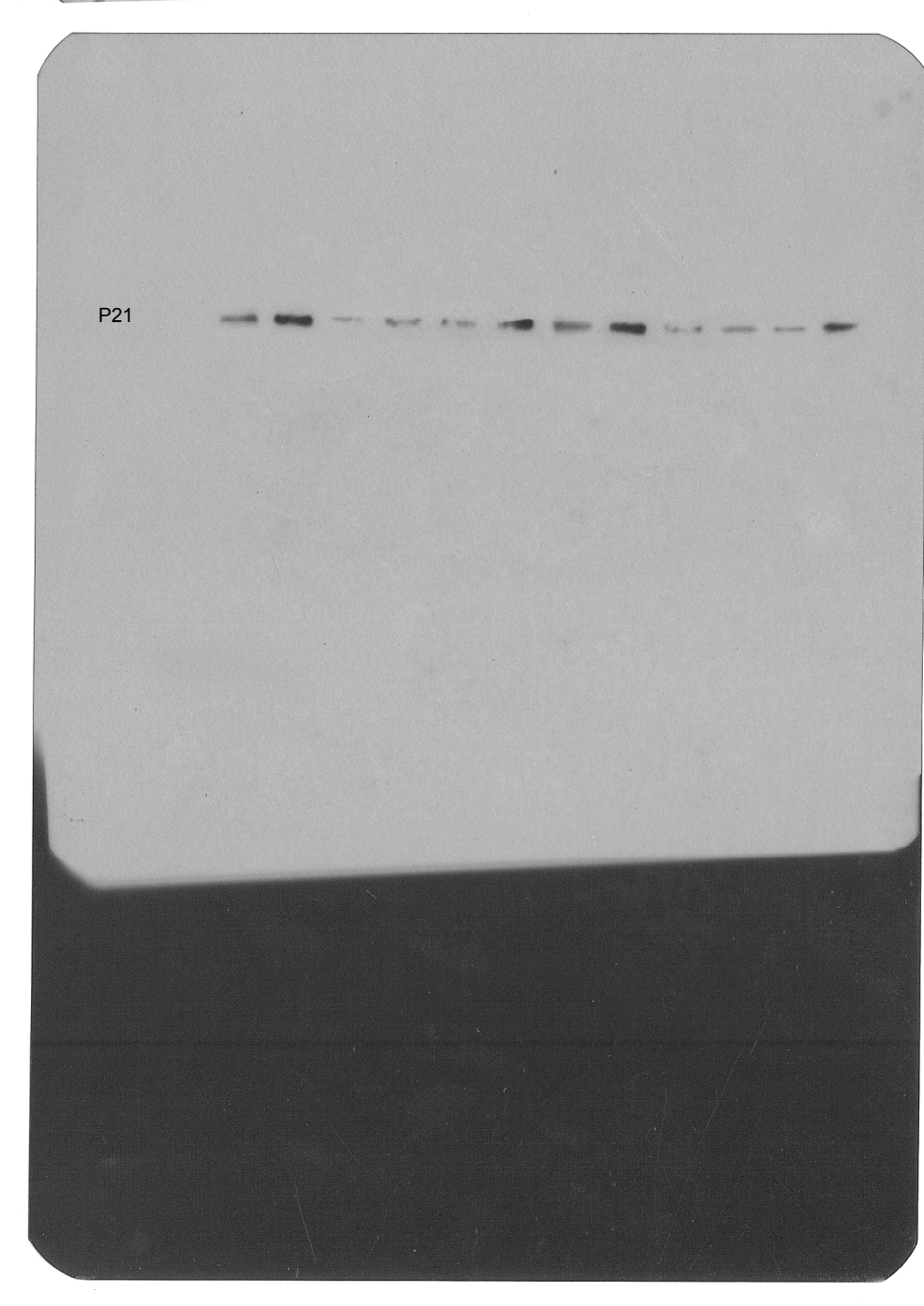

Supplement: Supplementary file 16 — WB_Fig.7N [file 41419_2022_5541_MOESM16_ESM.pdf]

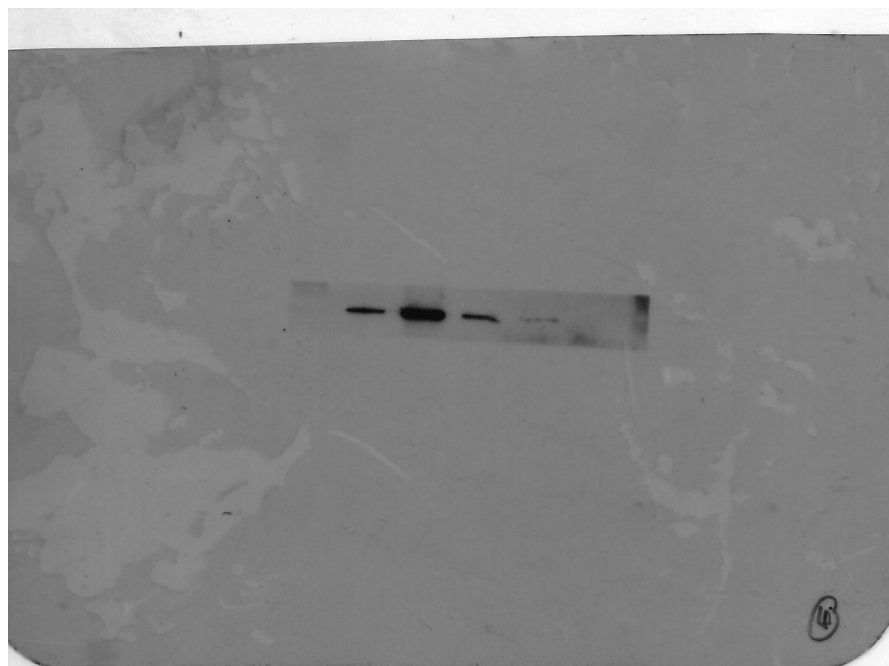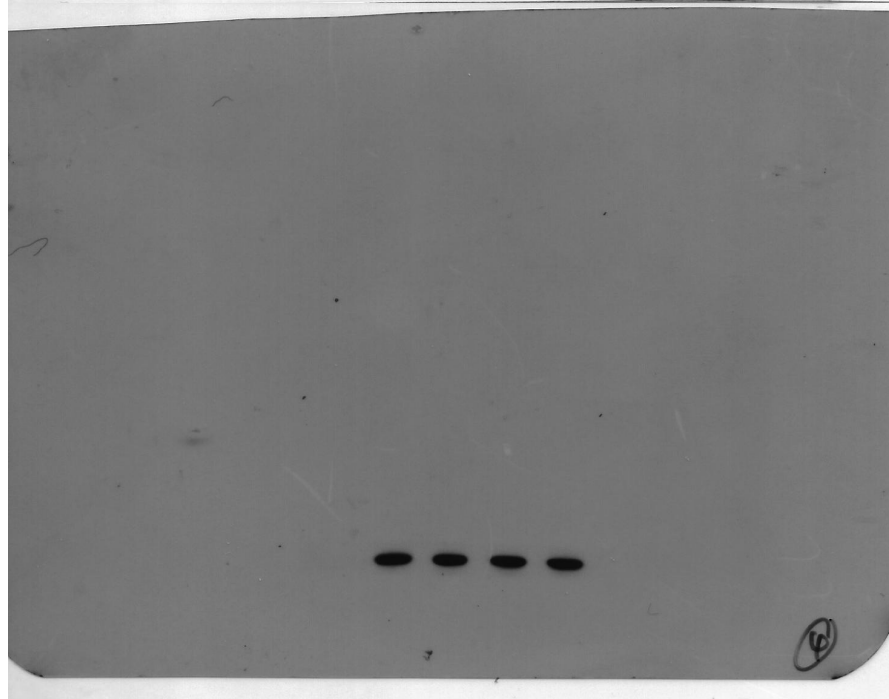

Supplement: Supplementary file 17 — WB_Fig.S3C [file 41419_2022_5541_MOESM17_ESM.pdf]

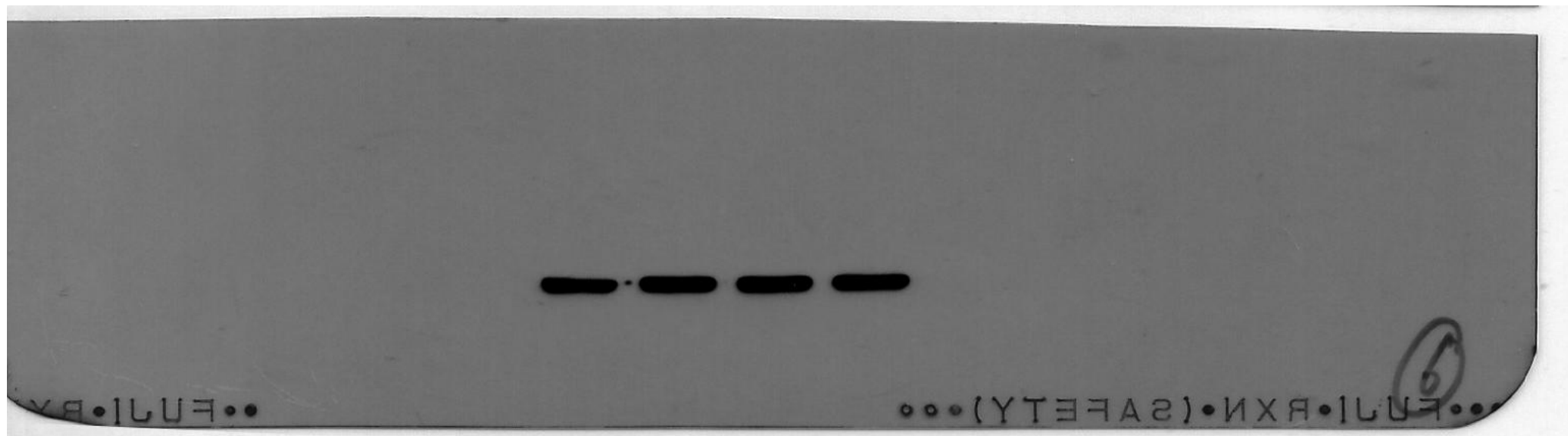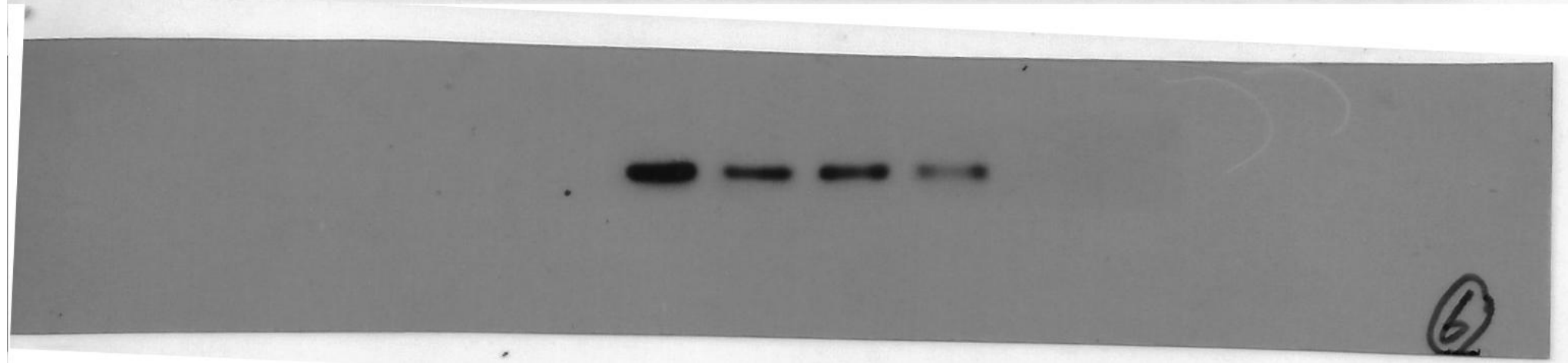

Supplement: Supplementary file 18 — WB_Fig.S4G [file 41419_2022_5541_MOESM18_ESM.pdf]

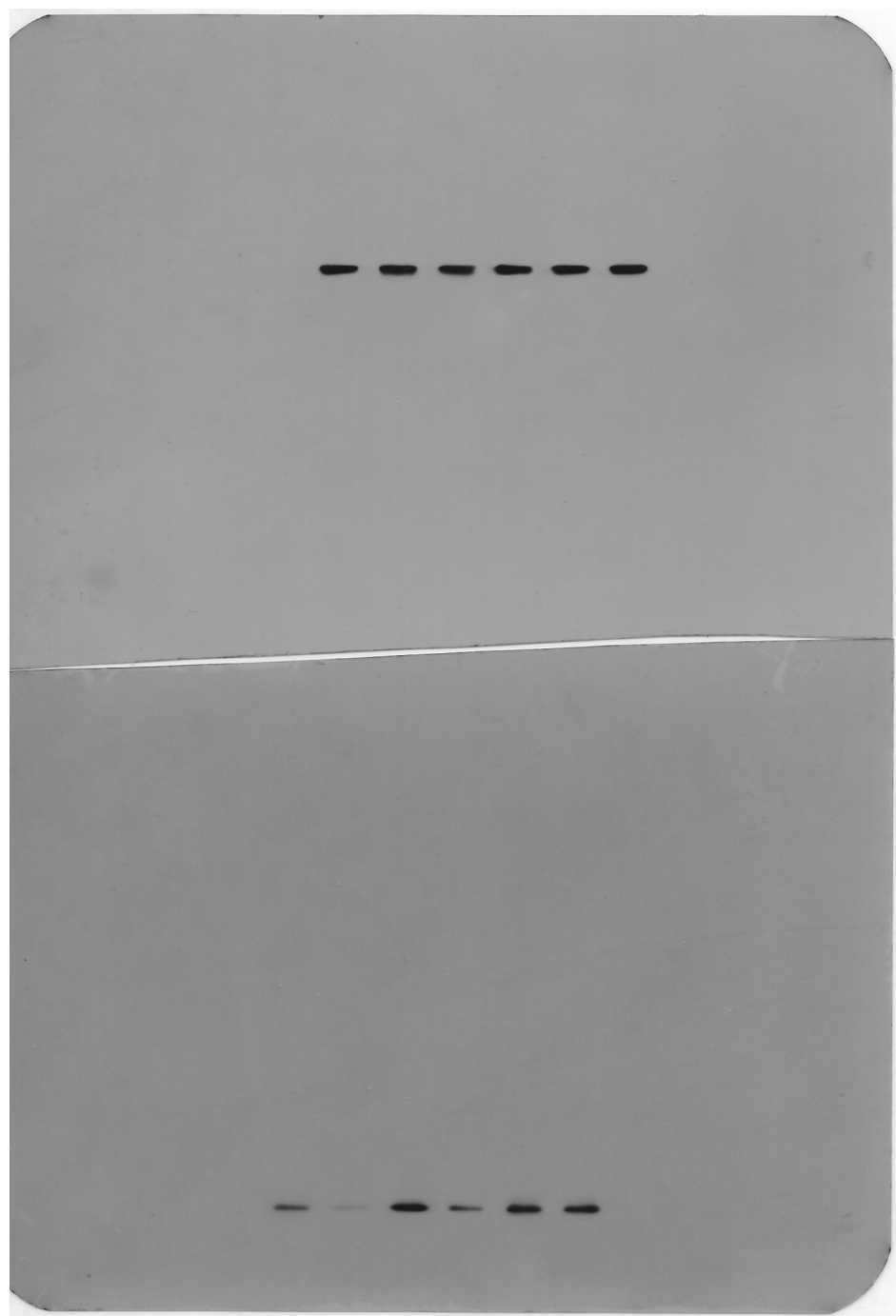

Supplement: Supplementary file 19 — WB_Fig.S7 [file 41419_2022_5541_MOESM19_ESM.pdf]
